# Geonomics: Forward-Time, Spatially Explicit, and Arbitrarily Complex Landscape Genomic Simulations

**DOI:** 10.1093/molbev/msab175

**Published:** 2021-06-12

**Authors:** Drew E Terasaki Hart, Anusha P Bishop, Ian J Wang

**Affiliations:** Department of Environmental Science, Policy, and Management, College of Natural Resources, University of California, Berkeley, CA, USA

**Keywords:** landscape ecology, evolutionary genetics, population dynamics, environmental change, spatial modeling, Python

## Abstract

Understanding the drivers of spatial patterns of genomic diversity has emerged as a major goal of evolutionary genetics. The flexibility of forward-time simulation makes it especially valuable for these efforts, allowing for the simulation of arbitrarily complex scenarios in a way that mimics how real populations evolve. Here, we present Geonomics, a Python package for performing complex, spatially explicit, landscape genomic simulations with full spatial pedigrees that dramatically reduces user workload yet remains customizable and extensible because it is embedded within a popular, general-purpose language. We show that Geonomics results are consistent with expectations for a variety of validation tests based on classic models in population genetics and then demonstrate its utility and flexibility with a trio of more complex simulation scenarios that feature polygenic selection, selection on multiple traits, simulation on complex landscapes, and nonstationary environmental change. We then discuss runtime, which is primarily sensitive to landscape raster size, memory usage, which is primarily sensitive to maximum population size and recombination rate, and other caveats related to the model’s methods for approximating recombination and movement. Taken together, our tests and demonstrations show that Geonomics provides an efficient and robust platform for population genomic simulations that capture complex spatial and evolutionary dynamics.

## Introduction

Spatial patterns of genomic diversity result from the complex interplay of many underlying ecological and evolutionary processes and are shaped by a wide variety of geographic and environmental factors. Understanding how these patterns develop in natural systems has emerged as a primary goal of modern evolutionary genetics. These systems often occupy complex and potentially changing landscapes and might include populations that are not at demographic equilibrium. They may undergo neutral evolution as well as natural selection, sometimes on multiple traits of variable genetic architecture. The study of complex natural systems is crucial for developing evolutionary and ecological theory (Epperson et al. 2010; Barrett et al. 2019; Pelletier 2019), understanding the forces governing the evolution and maintenance of genetic diversity (Manel et al. 2003; Schoville et al. 2012), anticipating ecological futures in the Anthropocene (Bay et al. 2018; Capblancq et al. 2020), and informing conservation and management (Crossley et al. 2017; Lind et al. 2017). The complex genomics of many such systems are beyond the reach of analytical population genetics, and their spatial complexity and evolutionary dynamics make them intractable for coalescent simulation (Hoban et al. 2012). This hinders not only our understanding of many empirical systems but also our ability to predict their dynamics and, thus, to manage them. Hence, in population and landscape genomics, as in many other fields, forward-time simulation is a crucial tool for dissecting complex study systems.

However, the current suite of forward-time genomic simulators, although numerous, is still of limited utility for such work. Most available software is restricted, either genomically or geospatially, in the complexity, it can model. Many programs can model systems of considerable genomic complexity (e.g., simuPOP, Peng and Kimmel 2005; NEMO, Guillaume and Rougemont 2006; QuantiNemo, Neuenschwander et al. 2008) yet incorporate only rudimentary spatial components or none at all. Other programs are designed specifically for landscape genetic simulations (e.g., CDPOP, Landguth and Cushman 2010; CDMetaPOP, Landguth et al. 2017; SimAdapt, Rebaudo et al. 2013) but are limited in their genomic complexity. For instance, many programs are unable to model simultaneous selection on multiple, polygenic traits. To our knowledge, SLiM (Messer 2013; Haller and Messer 2017; Haller and Messer 2019) is the only package currently capable of simulating scenarios that are sufficiently complex, both genomically and geospatially, to model population genomic patterns emerging under dynamic evolutionary processes (according to a search of the National Cancer Institute’s Genetic Simulation Resources website; Peng et al. 2013), and its extreme generalizability and complexity allow it to be used for landscape genomics simulation. Furthermore, many species are distributed continuously in space, and examining continuous fields of genetic variation can require distinct methods and assumptions (Bradburd and Ralph 2019), yet most population genomic simulation packages, aside from SLiM, are population-based. Such software requires individuals to be assigned to discrete subpopulations, which can at best be arranged on a high-resolution, regular grid in order to approximate continuously distributed populations.

Here, we present Geonomics, a Python package for forward-time, individual-based, continuous-space, population genomic simulations on complex landscapes. Geonomics models are parameterized by way of an informatively annotated parameters file that provides the user a straightforward means of building models of arbitrary complexity while offering reasonable default settings and “off switches” for parameters and components unrelated to the user’s interests. Models consist of 1) a landscape with one or more environmental layers, each of which can undergo arbitrarily complex environmental change events and 2) one or more species having genomes with realistic architecture and any number of associated phenotypes. Species undergo non-Wright-Fisher evolution in continuous space, with localized mating and mortality, such that species-level phenomena and simulation dynamics are emergent properties of a model’s parameterization. Evolution is comprehensively tracked by way of recently developed data structures that record the complete spatial pedigree (Kelleher et al. 2018), providing for the customizable output of rich, 3D data sets in a variety of common formats, including VCF and FASTA for genomic data, GeoTiff for landscape data, and CSV, Shapefile, and GeoJSON for individuals’ nongenomic data (location, environmental values, phenotypes, age, and sex). All of this allows Geonomics to produce realistic landscape genomic results useful for a wide variety of theoretical and empirical purposes.

## New Approaches

### Model Design: Overview

A Geonomics model consists of two core components: the species and the landscape. The species is composed of a set of individuals and a wide variety of demographic and life-history parameters, including an intrinsic growth rate, mate-search radius, the mean number of offspring per mating event, reproductive age, and maximum age, among others. A species can undergo any number of change events, including changes to demographic and life history parameters and various types of population size changes. Each individual in the species has an *x, y* location, a sex, an age (or life-history stage), a set of phenotypes and a diploid genome consisting of any number of diallelic loci, which can represent either a contiguous haplotype block or a set of distinct loci. Loci can exhibit different types of dominance, and recombination rates can be heterozygous across the genome.

Phenotypic traits are continuous and quantitative and can be monogenic or multigenic. Each trait is defined by the loci that comprise its genetic basis, the effect sizes of those loci, and a phenotypic selection coefficient, which can be made heterogeneous in both space and time, allowing for spatially complex selection scenarios. While the strength of selection is determined by that coefficient, the force of selection is represented by the environmental raster layer to which the trait is adapting. Loci can have separate mutation rates for three types of mutations: neutral, deleterious, and trait-affecting mutations. Neutral mutations do not affect fitness, and deleterious mutations decrease fitness without affecting simulated phenotypic traits. Trait-affecting mutations, on the other hand, introduce mutations at previously unmutated loci mapped to a trait. This adds to the genetic variation affecting a trait, thus generating phenotypic variance upon which natural selection can operate. Mutation rates can be defined separately for each trait.

The other core component of a Geonomics model, the landscape, is a stack of raster layers. Each layer can be set to serve as one or more of 1) a resistance raster, which controls individual movement or offspring dispersal, 2) a carrying-capacity raster, which controls population density, and 3) a fitness raster for a trait, which governs natural selection. A key feature of Geonomics is that each layer can undergo any number of arbitrarily complex environmental change events which, as they unfold, influence the dynamics of any species whose carrying capacity, movement and dispersal, or fitness depend on the corresponding layer.

### Model Operation: Overview

**Fig. 1.**
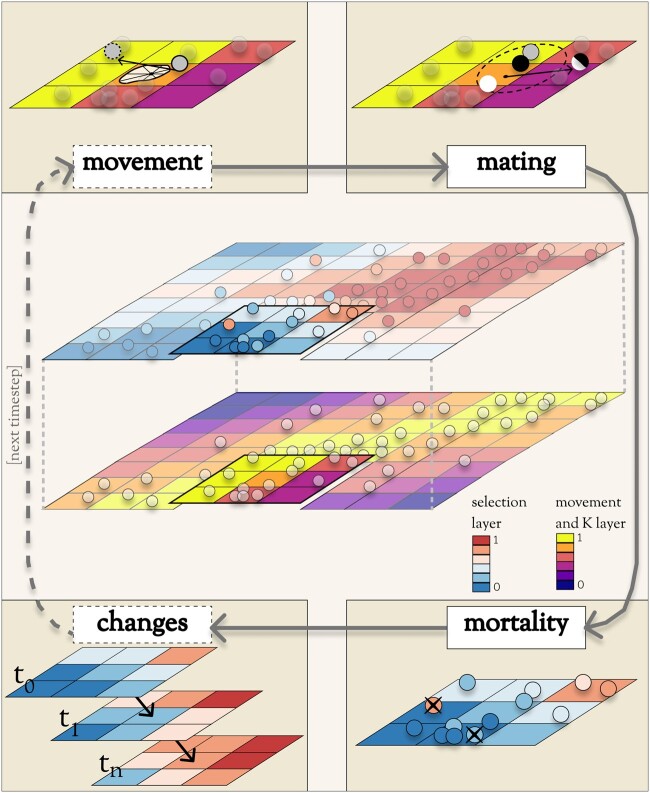
Operations during the main phase of a Geonomics model run. In the center is a species on a multilayer landscape that includes a selection layer (above) and a layer for movement and carrying capacity (below). Surrounding the landscape is a flow-diagram of the major operations during a time step. Operations in dashed boxes are optional. During the movement stages (top-left), individuals move along movement vectors drawn from various distribution options (shown is an example of a cell-specific von Mises mixture distribution). During the mating stage (top-right), each mating individual (black circle) randomly chooses a mate (white circle) from all potential mates within its mating radius (dashed circle). The resulting offspring (half-black, half-white circle) disperses from its parents' midpoint along a randomly drawn dispersal vector. During the mortality stage (bottom-right), deaths are modeled as a Bernoulli process, with the probability of mortality a product of density-dependence and selection on all traits. During the changes stage (bottom-left), environmental and demographic change events, which can be represented by a series of change rasters corresponding to scheduled time steps, take place.

A Geonomics run begins with a burn-in stage during which individuals move and reproduce, without genomes or selection, until a series of statistical tests is passed. These tests include a time-lagged *t*-test and an augmented Dickey–Fuller test, which are run as a pair for 1) the total population size, serving as a test of temporal demographic stability, and 2) both the mean and the standard deviation of timestep-differenced cell-wise counts of individuals, serving as a test of spatial demographic stability. This burn-in period results in a stationary spatial distribution of the species on the landscape. Following burn-in, each individual has its genome randomly assigned according to the genomic architecture parameters, such that the main phase of each run begins with no pedigree and, thus, without population structure. Each time step in the main phase is a series of four operations, some optional (fig. 1):

1. movement (optional);
2. mating (requisite), which includes mate search, mate choice, offspring creation, and offspring dispersal;
3. mortality, which is due to density-dependence (requisite) and natural selection (optional);
4. change events (optional), including both environmental and demographic changes.

### Model Operation: Movement

**Fig. 2.**
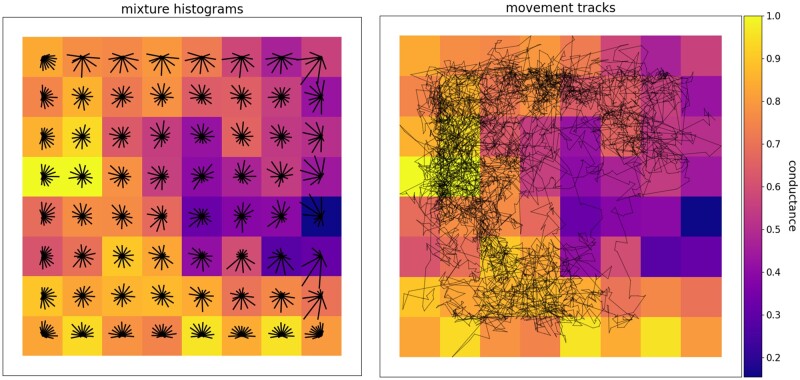
A raster layer representing a movement surface with example movement histograms for each cell (left) and a movement track for a sample individual (right). The circularized histograms represent the movement directions that could be drawn from the von Mises mixture distribution approximations within each cell. Longer bars in a histogram indicate higher probability of movement in their direction. The movement track, plotted with the *gnx.help.param_help.plot_movement* function in Geonomics, is 5,000 steps long. Both preferential movement toward higher-suitability regions of the landscape (i.e., cells closer to 1 in value) and occasional long-distance movements between relatively isolated portions of the landscape are evident.

Movement takes place in continuous space—individuals have *x, y* coordinates, on either real or simulated landscapes, rather than being arbitrarily restricted to grid cells or bounded populations. Each individual moves along a vector, composed of a distance drawn from a Wald distribution and a direction drawn either from a uniform distribution on the unit circle or from a movement surface—an array of unimodal or multimodal von Mises distributions derived from a landscape layer that serves as a resistance surface (sensu McRae 2006; Spear et al. 2010). On a unimodal movement surface, each cell is assigned a single von Mises distribution, with mode parameter μ set to the direction of the highest-valued cell in the 8-cell neighborhood. On a multimodal surface, each cell’s mixture distribution is a weighted sum of eight such unimodal distributions, one pointing toward the center of each cell in the 8-cell neighborhood and with normalized weights equal to the values of the neighboring cells. This approach to simulating movement generates realistic, anisotropic movement across a heterogeneous landscape (fig. 2) while avoiding time-consuming computational steps, such as repeated searches for minimum-resistance neighboring cells.

### Model Operation: Mating

Potential mating pairs are randomly drawn from among all eligible pairs of individuals within the mate-search radius (unless strict nearest-neighbor mating is chosen), with pairing probabilities either uniform or inverse-distance weighted within the mating radius, and with eligibility based on both sex and age. From among those pairs, actual mating-event decisions are Bernoulli distributed, with probability equal to the intrinsic birth rate. Each mating pair produces a number of offspring according to a fixed value or drawn from a Poisson distribution with λ equal to the mean number of offspring. Each parent produces a gamete for each of its offspring, using realistic recombination and Mendelian segregation. Gametes are united to create offspring individuals, which then disperse to new locations. As with movement, dispersal vectors can be drawn isotropically or anisotropically based on a resistance surface.

### Model Operation: Mortality

Mortality is modeled as a Bernoulli process with the probability of an individual death a combination of the probabilities of death due to density-dependence (using a logistic-growth model) and due to natural selection (based on the cumulative fitness for all traits), calculated as:

(1) $$P\left(d_{i}\right)=1-\left(1-P\left(d_{x,y}\right)\right)\prod_{p=1}^{m}\omega_{i,p},$$

where $P\left(d_{x,y}\right)$ is the probability of death due to density-dependence for individual *i*, *m* is the number of traits, and $\omega_{i,p}$ is the fitness of individual *i* for trait *p*. The probability of density-dependent death at location *x,y* is calculated as:

(2) $$P\left(d_{x,y}\right)=\frac{E\left[N_{d;x,y}\right]}{N_{x,y}}=\frac{E\left[N_{b;x,y}\right]-\frac{dN_{x,y}}{dt}}{N_{x,y}},$$

where, for location *x,y*, $E\left[N_{d;x,y}\right]$ is the expected number of deaths,$\;N_{x,y}$ is the population density, $E\left[N_{b;x,y}\right]$ is the expected number of births, and $\frac{dN_{x,y}}{dt}$ is the population logistic growth rate. The fitness of individual *i* for trait *p* is calculated as:

(3) $$\omega_{i,p}=1-\phi_{p;x,y}{\left(|e_{p;x,y}-z_{i;p}|\right)}^{\gamma_{p}},$$

where $\phi_{p;x,y}$ is the phenotypic selection coefficient on trait p at location *x,y*, $e_{p;x,y}$ is the value of the selection layer for trait *p* at location *x,y*, $z_{i;p}$ is the phenotype of individual *i* for trait *p*, and γ_*p*_ defines the curvature of the fitness function for trait *p*. The phenotype is a result of the additive effects of that individual’s genotypes at all underlying loci, and is calculated as:

(4) $$z_{i;p}=\sum_{l=0}^{n}\alpha_{p,l}g_{i,l}+g_{0}$$

where n is the number of loci, $\alpha_{p,l}$ is the effect size of locus *l* on trait *p*, $g_{i,l}$ is the genotype at locus *l* for individual *i*, and $g_{0}$, the baseline genotype, equals 0 for monogenic traits or 0.5 for polygenic traits.

### Model Operation: Change Events

Each demographic or environmental change event unfolds as a series of incremental changes that occur at the ends of scheduled time steps. A demographic change event can be exponential, random, or cyclical, or it can follow an arbitrarily complex, custom trajectory. Each event is parameterized by defining the time steps at which its changes take place and the factor by which the carrying capacity raster is multiplied.

Simple environmental change events are defined by a terminal raster for the final environmental state and a list of time steps at which incremental changes occur (based on cell-wise linear interpolation between the beginning and terminal states). More complex, custom events can be simulated by providing a series of environmental rasters labeled with the time step at which each will be applied. This option makes it easy to simulate evolution on real-world landscapes undergoing nonlinear, spatially heterogeneous environmental change.

## Results

### Validation

To validate the performance of Geonomics, we ran a series of simulations based on classical population genetic models, covering both neutral and nonneutral evolutionary scenarios. Because the classical models are simpler than the individual-based, spatially explicit, continuous-movement models built by Geonomics, we parameterized the simulations so as to accurately emulate these models while minimizing artifacts (see Validation Testing, Supplementary Material online). Our goal was to statistically and heuristically validate Geonomics’ full range of functionality and to ensure that it accurately models neutral and nonneutral evolutionary processes.

To verify that Geonomics effectively models neutral evolution, we first examined the average time to fixation for a neutral allele in a finite population using simulations approximating a Wright-Fisher model (Fisher 1923; Wright 1930). We simulated allele-frequency trajectories for 250 independent loci (25 of which are plotted in supplementary fig. S1, Supplementary Material online), and we found that that fixation time did, indeed, increase with population size and was proportional to 4N_e_, as expected (Kimura and Ohta 1969), in our simulations (supplementary fig. S2, Supplementary Material online). We then tested for changes in the rate of drift surrounding a population bottleneck event by forcing a population to undergo a 70% reduction in size for 50 of 300 timesteps. We found that rates of allele frequency change increased during the bottleneck, then returned to prior levels shortly thereafter (supplementary fig. S3, Supplementary Material online). Finally, we quantified the accumulation of genetic structure under a stepping-stone model (Kimura 1953) to certify that genetic covariance decreases with distance (Kimura and Weiss 1964). As expected, we saw that migration rates decreased as a function of inter-island distance (supplementary fig. S4, Supplementary Material online), whereas F_ST_, calculated from both heterozygosity data and genetic variance data, increased as a function of inter-island distance (and, therefore, decreased with pairwise migration rate) and as a function of time (supplementary figs. S4 and S5, Supplementary Material online). We also performed a discriminant analysis of principal components (DAPC) using the R package *adegenet* (Jombart et al. 2010) to confirm the expected population structure of six island clusters (supplementary fig. S6, Supplementary Material online).

To validate the performance of Geonomics for modeling nonneutral evolution, we first performed simulations under a simple scenario of divergent selection between two discrete habitats. As expected, simulations on a landscape evenly divided by two habitat blocks led to local adaptation, producing a significant pattern of phenotype-habitat matching (supplementary fig. S7, Supplementary Material online), with mismatches concentrated along the border between habitats. Additionally, over time, the species reached migration–selection equilibrium—the frequencies of the beneficial alleles in each habitat increased up to a stationary level, with that level being positively correlated with the strength of selection (supplementary fig. S8, Supplementary Material online). A plot of the mean difference between individuals’ phenotypic and environmental values shows a strong decline over model time, with the rate and level of decline increasing as a function of increasing strength of selection (supplementary fig. S9, Supplementary Material online). Finally, logistic regressions show no significant relationships between phenotypic and environmental values at the outset (pseudo-*R*^2^s ≈ 0.0, *P* values > 0.1), but show highly significant relationships at the ends of the simulations (*P* < 0.0001 for all values of Φ), with the amounts of variation explained increasing as a function of selection strength (pseudo-*R*^2^ = 0.327 for Φ = 0.01, 0.376 for Φ = 0.05, and 0.406 for Φ = 0.1).

We next tested the ability of Geonomics to recreate the genetic structure expected under local adaptation along an environmental cline: monotonic change in the allele frequency of a nonneutral locus across the cline. On a landscape with a symmetric environmental selection gradient, Geonomics again produced the expected spatial pattern of local adaptation (supplementary fig. S10, Supplementary Material online), and when we fitted sigmoid *tanh* clines (Szymura and Barton 1986; Porter 2013) for all loci, the locus underlying the monogenic trait was the only one to exhibit clinal variation (supplementary fig. S11, Supplementary Material online). In a family of genotype-environment analyses using Bonferroni-corrected, locus-wise logistic regressions, this locus was also the most significantly correlated with the environmental variable based on locus-wise logistic regressions (*P* < 0.0001). A plot of the mean difference between individuals’ phenotypic and environmental values shows a strong decline over model time (supplementary fig. S12, Supplementary Material online), and logistic regressions show no significant relationship between phenotypic and environmental values at the outset (pseudo-*R*^2^ = 0, *P* value = 0.812) but a significant relationship at the end of the simulation (pseudo-*R*^2^ = 0.169, *P* value < 0.0001).

Finally, we verified that Geonomics can effectively model genomic data with physical linkage by simulating a selective sweep, introducing a beneficial mutation at the center of a 101-locus block of otherwise neutral loci. The results exhibited the classic genomic signal of a selective sweep (Przeworski 2002; Kim and Nielsen 2004), with a region of reduced nucleotide diversity surrounding the locus under selection and that region gradually eroding over time (supplementary fig. S12, Supplementary Material online). During these simulations, as the beneficial mutant spread through the population the population’s mean fitness increased from 1 − Φ (where Φ is the strength of selection) to a saturating value of 1 (supplementary fig. S14, Supplementary Material online), confirming that the population dynamics of the selective sweep played out as expected. To further support these results, we also validated Geonomics’ method of recombination by examining effective recombination rates observed in a Geonomics model to those produced by an msprime simulation using the same randomly drawn, heterogeneous recombination map (see Recombination Test, Supplementary Material online); the resulting genome-wide pattern of recombination breakpoint densities recapitulates the one produced by msprime and the true recombination map (supplementary fig. S15, Supplementary Material online).

### Example Applications: Overview

To demonstrate the broad utility of Geonomics for modeling complex evolutionary scenarios, we performed a series of simulations covering a range of potential applications. These demonstrations highlight scenarios for which Geonomics is particularly well suited, including spatially explicit simulations on highly heterogeneous landscapes, selection on multiple traits with complex genomic architecture, and microevolutionary responses to nonstationary environmental change.

### Example 1: Isolation by Distance and by Environment

Genetic covariances between individuals or populations are often inversely correlated with linear or resistance-based geographic distance—a pattern known as isolation by distance (IBD; Wright 1943) or isolation by resistance (IBR; McRae 2006; McRae et al. 2008)—or with environmental distance—a pattern known as isolation by environment (IBE; Wang and Bradburd 2014). Understanding the landscape factors and population processes generating these patterns has emerged as a major focus of landscape genetics (Sexton et al. 2014; Wang and Bradburd 2014).

To demonstrate how Geonomics can simultaneously generate patterns of IBD and IBE, we built a simulation that uses a heterogeneous resistance layer as a movement surface and models selection for a 10-gene trait on a heterogeneous environmental layer (*Φ* = 0.05). The model features a species with a stationary population size (roughly 2,450 individuals), experiencing both selection and neutral evolution. The resistance layer consists of a central barrier separating equal-area sides—the barrier has a high resistance to movement, but the movement is unconstrained on either side. This layer was also used as the carrying-capacity layer, yielding homogeneous population density on the two sides and zero density within the barrier region. The selection layer consists of two environmental gradients running in opposite directions on either side of the barrier, such that the landscape contains pairs of locations representing a range of combinations of geographic and environmental distances.

To observe the development of population structure, we collected data sets consisting of the genomes for all individuals at timesteps 0 and 1,000. We then used principal component analysis (PCA) to calculate pairwise genetic distances between all individuals for each data set. To visualize population structure, we extracted the first three principal components (PCs) and used them as the red, green, and blue (RGB) color values for mapping individuals on the landscape. To visualize the outcomes of selection, we produced paired maps of the same individuals colored by their phenotypes for the trait under selection (using Geonomics’ “model.plot_phenotype(…)” method), and also created a set of the population–structure plots using DAPC. To visualize the time course of the simulation, we plotted the mean phenotype–environment mismatch (i.e., the mean of |e-z|, the driving force of selection) and mean fitness. We visualized signals of IBD and IBE in the final data set using a 3D scatterplot of Euclidean pairwise genetic distance against Euclidean pairwise geographic and environmental distances, colored by pairwise phenotypic distances. We tested the significance of the relationship between genetic distance and environmental distance, controlling for geographic distance, using paired partial Mantel tests with the *vegan* package (version 2.5-6; Oksanen et al. 2019) and using multiple matrix regression (MMRR; Wang 2013) in R version 4.0.2 (R Core Team 2020). Finally, because Geonomics models do not use defined landscape-resistance values, we quantified the barrier’s increased landscape resistance by tracking all barrier-crossing events, using them to calculate the per time step crossing rate, then comparing that to the equivalent crossing rate of the same landscape zone in an otherwise identical model that omitted the barrier.

**Fig. 3.**
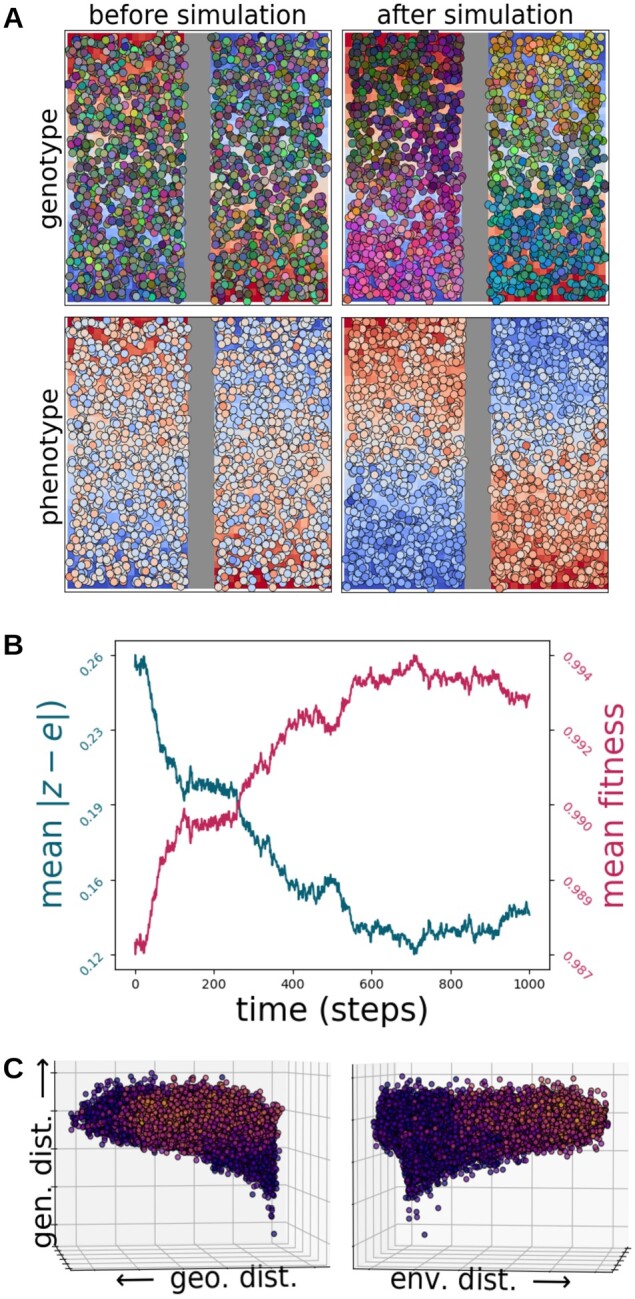
Results of simulations for the isolation by distance (IBD) and isolation by environment (IBE) example application, in which a species evolved on a landscape with a barrier layer that served as the movement surface (displayed as a vertical gray band down the landscape) and an environmental layer that served as the selective surface for a 10-locus trait (displayed as the red to blue gradient on the landscape). (*A*) The population before the simulation (left column) and after it (right column), colored by genetic distance (top row), with colors derived from scores on the first three PCs of a genetic PCA used to assign RGB values, and by phenotype (bottom row). The most-fit individuals are those whose phenotypic colors perfectly match the cells on which they are located. (*B*) The time courses of the mean difference between individuals’ phenotypes and their environmental values (blue) and of mean fitness values (red). (*C*) Two views of a 3D scatter plot of pairwise genetic distance as a function of Euclidean geographic distance (left) and Euclidean environmental distance (right), with points colored by phenotypic distance.

The RGB and phenotype plots of the initial population, with randomly assigned genomes, showed a clear lack of both spatial structure and local adaptation (fig. 3, top left). However, as expected, spatial structure developed over time, and the species showed signs of local adaptation over the course of the simulation (fig. 3, top right), as well as a corresponding, hierarchical population structure (supplementary fig. S15, Supplementary Material online). Average phenotype-environment mismatch decreased and average fitness increased over time (fig. 3, top middle). At the end of the simulation, the species demonstrated significant signals of both IBD (partial Mantel test: *r* = 0.560, *P* ≤ 0.001; MMRR: *P* ≤ 0.001) and IBE (partial Mantel test: *r* = 0.121, *P* ≤ 0.001; MMRR: *P* ≤ 0.001; MMRR full model *R*^2^ = 0.354), as evidenced by the positive slopes on both horizontal axes of the 3D scatterplot (fig. 3, bottom). The colors of the points in the 3D scatter plot also indicate a clear pattern of increasing phenotypic differences between individuals with increasing environmental distance (fig. 3, bottom right) but not between individuals separated by increasing geographic distances (fig. 3, bottom left). Finally, the barrier zone had an observed crossing rate of 0.004 individuals per time step in this model, 13 times lower than the rate of 0.052 individuals per time step observed in the barrier-less but otherwise identical model. These results show that Geonomics effectively models IBD and IBE, driven by divergent natural selection, using two simple raster layers. More complex layers could be used to simulate IBD and IBE under a wide range of scenarios, and empirical layers could be used to simulate patterns of spatial genetic variation on real-world landscapes.

### Example 2: Simultaneous Election

One of the most powerful features of Geonomics is that it can simulate selection on numerous traits simultaneously, each responding to a separate selection layer. Thus, a simulated species can experience multiple spatial selection regimes. Many natural systems are locally adapted to multiple environmental variables (Fournier-Level et al. 2011; Lasky et al. 2012; Manel et al. 2012), so simulating these scenarios could be broadly valuable for investigating the nature of local adaptation in real environments.

**Fig. 4.**
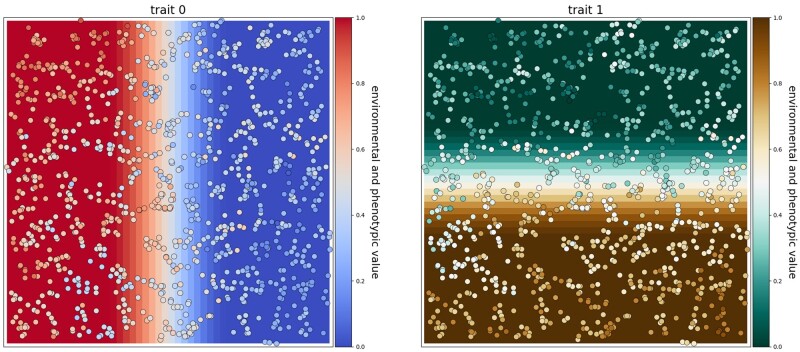
Results of simultaneous selection on two traits with spatially distinct selective regimes. Each trait is controlled by 10 unlinked loci and has a selection coefficient of Φ = 0.05. Individuals are colored by phenotype for the trait under selection on each layer.

To demonstrate how Geonomics can model simultaneous selection, we simulated a scenario in which a species undergoes natural selection along two orthogonal environmental gradients, each driving selection for a separate trait (Φ = 0.05). Each trait had values ranging from 0 to 1, determined by 10 loci, all with equal effect sizes. Individuals had a mean movement distance of 0.5 cell widths on a 50 × 50-cell landscape, chosen to limit gene flow and allow for the development of strong spatial structure and, thus, the potential for local adaptation. We let the system evolve for 1,000 time steps and then mapped the species on each of the environmental layers, with individuals colored by phenotype in order to visually evaluate whether individual phenotypes matched their environmental backgrounds. The results showed clear patterns of phenotype–environment matching along both independent gradients (fig. 4) that evolved steadily through time (supplementary fig. S16, Supplementary Material online; compare to supplementary figs. S17 and S18, Supplementary Material online, with Φ = 0), indicating strong evidence for simultaneous selection across the simulated landscape.

### Example 3: Polygenic Adaptation to Climate Change in the Yosemite Region

Better understanding evolutionary responses to changing environments is essential for predicting species outcomes and preserving biodiversity under ongoing climate change (Hoffmann and Sgrò 2011; Franks and Hoffmann 2012; Bay et al. 2018; Capblancq et al. 2020). In many regions, climate shifts are projected to be spatially heterogeneous, including in montane regions where cooler, higher-altitude areas are warming more quickly than warmer, low-altitude regions (Rangwala et al. 2013; Mountain Research Initiative EDW Working Group 2015; but see Oyler et al. 2015). Of particular interest under these scenarios is the ability of species to adapt to changing local conditions (Franks and Hoffmann 2012).

To demonstrate the utility of Geonomics for studying microevolutionary responses to climate change, we simulated the response to projected climate change of a continuously distributed, locally adapted species, using the sagebrush lizard (*Sceloporus graciosus*) in the topographically complex Yosemite National Park region of California (USA) as an empirical model. To model climate change, we assembled time series raster stacks of projected mean annual temperature, annual precipitation, and habitat suitability for 19 even time steps from the present through the year 2100. For present temperature and precipitation, we used PRISM data (Daly et al. 2008), calculated as 30-year normals for 1981–2010 at 800 m resolution. For future years, we used means at a set of 5-year intervals (2015–2100), downscaled to 6 km resolution using the localized constructed analogs downscaling technique (LOCA; Pierce et al. 2014), from the Cal-Adapt database (https://cal-adapt.org/). We calculated means of both variables from their minima and maxima observed across 32 global climate models, using a conservative representative concentration pathway (RCP 4.5). We developed time series of future temperature and precipitation layers at 800-m resolution by 1) calculating the raster difference between the first projected year and the current data, aggregated to the projected data’s resolution; 2) adding that difference to the current data, such that each cell in the current data received the difference of the coarser, projected cell within which it lay; and 3) repeating that process for all remaining years. All data preparation was done using custom scripts (Supplementary Material online) in R (R Core Team 2020). 

For the habitat suitability rasters, we constructed a species distribution model (SDM) using the present-day temperature and precipitation variables. We downloaded all georeferenced *S. graciosus* occurrence data from the Global Biodiversity Information Facility database (www.gbif.org), using the *gbif* function in the *dismo* R package (Hijmans et al. 2017). We clipped the points to California and Nevada, then subsampled the full data set to remove multiple points within the same raster cells. We generated pseudoabsence data by drawing random points from all cells in the California-Nevada region where the species was not observed (following the recommendations of Barbet‐Massin et al. 2012). We extracted the current temperature and precipitation data at these points and used them as predictor variables in a binomial generalized linear model (GLM) with a logit link. We then projected that GLM onto the current and future temperature and precipitation rasters for our study region, producing a time series of predicted habitat suitability. 

We generated the simulation’s parameters file using the code provided in Code Sample S1, then edited the parameter values therein as needed. To simulate the nonneutral evolution of a polygenic, quantitative trait, we set the trait to be underlain by 100 loci randomly distributed across a genome of 1,000 loci and set a strength of selection of Φ = 0.5. We set other life-history and demographic parameters (carrying capacity, age at reproductive maturity, number of offspring per individual, and maximum age) to reasonable values based on *S. graciosus* natural history (Stebbins 1948; Tinkle 1973; Rose 1976; Ruth 1978; Tinkle et al. 1993; Supplementary Material online).

We ran the main phase for 500 time steps without climate change (to develop a pattern of local adaptation), then ran an additional 100 time steps (years) with changing climate (see Code Sample S2). At time steps 500 (before the initiation of climate change events) and 600 (after completion of climate change events), we plotted the current temperature and habitat-suitability landscape layers along with a kriged surface of the current population’s phenotypes and a kernel density map of the current population’s density, two key emergent properties of the model that should be driven by temperature and habitat suitability, respectively. We then ran the model for an additional 50 timesteps to be able to more clearly visualize the effect of climate change on population size.

**Fig. 5.**
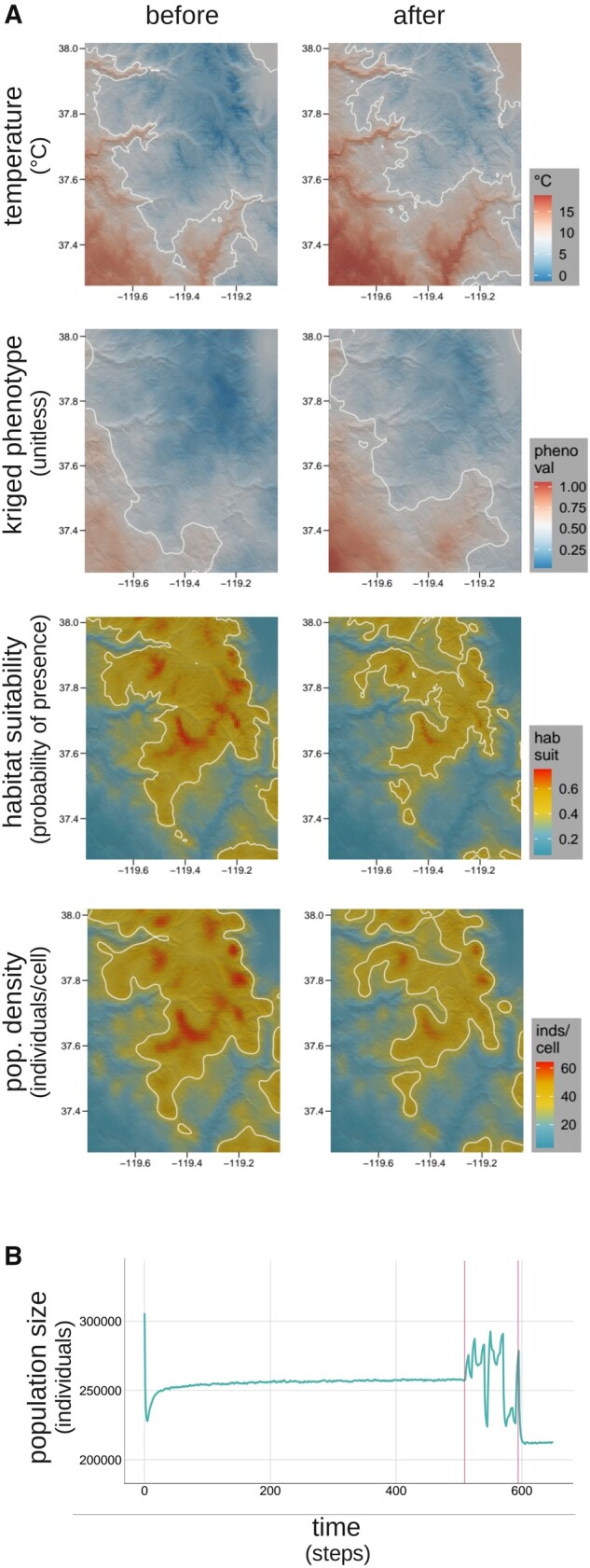
Polygenic adaptation to climate change in the Yosemite region. (*A*) Hillshade plot comparisons of key variables (mean temperature, phenotype, habitat suitability, and population density) before and after the simulated climate change event. The mean of each variable through time is used to draw midvalue contours on each map (white lines) to help visualize spatial change. As expected, the spatiotemporal shift in temperature (first row) drives a spatially corresponding shift in phenotypes, visualized as a surface kriged from all phenotypic values (second row), and the shift in habitat suitability (third row) likewise drives a corresponding shift in population density, visualized using a 2D kernel density estimator (fourth row). (*B*) The time course of population size. The early drop in population size results from the onset of natural selection after completion of the unplotted burn-in portion of the model. The oscillations and ultimate reduction at the end of the simulation are a result of the climate change event, which occurs during the period bracketed by vertical red lines.

The model generated a clear and realistic pattern of adaptation to the spatial temperature gradient in the Yosemite region after the 500 iterations following burn-in, and that pattern demonstrated a spatial shift in phenotypes that aligns clearly with the spatial shift in temperature under the simulated climate change scenario (fig. 5*A*, rows 1 and 2; Video 1, Supplementary Material online). The model also generated a spatial pattern of population density that clearly aligns with spatial variation in habitat suitability prior to the onset of climate change that likewise shifted as expected in response to the climate change-induced shift in habitat suitability (fig. 5*A*, rows 3 and 4). We observed demographic changes in response to climate change over the course of the simulation as well. After climate change, mean population size was reduced by roughly 16.8% (from about 255,500 to 212,500 individuals), in line with the 17.9% reduction in the carrying capacity layer derived from the habitat suitability rasters (from 337,089.0 to 276,742.8 individuals, according to sums of the pre- and post-change carrying capacity layers). The population also exhibited sizable fluctuations during the climate change period, with oscillations exceeding 60,000 individuals (roughly 23.5% of the pre-change mean population size; fig. 5*B*). We interpret this as a result of the stepwise environmental changes comprising the climate change event. Each change causes a shift in the optimum phenotypes of local populations, leading to increased maladaptation and thus increased mortality rates. Subsequent reductions in density-dependent mortality rates because of these reduced population densities, paired with adaptation by natural selection, then reduce overall mortality rates, leading to rebounds in population size, with stochastic movement into and out of local populations, along with other sources of model stochasticity, imposing noise on this oscillatory behavior. Overall, these results show how Geonomics can effectively simulate organismal responses to highly complex environmental scenarios and reveal that these simulations can uncover system behavior that could provide avenues for future investigation.

## Discussion

Our validations tests demonstrate that Geonomics simulates molecular evolution in concordance with predictions from theoretical population genetics (Fisher 1923; Wright 1943; Kimura; Szymura and Barton 1986), including dynamics of genetic drift, migration, and selection along clines, and our example applications show that Geonomics is capable of generating accurate and realistic population and landscape genomic data sets under scenarios of varying complexity. Geonomics is embedded in Python (van Rossum 1995; Python Software Foundation 2019), one of the most popular programming languages and one already familiar to many researchers who use bioinformatics. It makes the creation of arbitrarily complex models quick and easy, without even requiring prior Python experience, yet provides advanced users with access to core data structures, enabling broad customization and extension.

Many theoretical questions in population genomics necessitate explicitly spatial study methods, often with full tracking of a population’s spatial pedigree (Bradburd and Ralph 2019). Geonomics makes this work more tractable than ever before. Landscape genomics studies draw conclusions about complex, real-world systems, sometimes with direct implications for conservation and management (Epperson et al. 2010; Landguth et al. 2012). Geonomics not only enables the generation of simulated data sets specific to such study systems but will also aid the development and testing of analytical methods in landscape genomics, strengthening our ability to draw accurate and reliable inferences from real-world data.

### Runtime and Memory

Geonomics models run more slowly and have steeper memory limitations than models written and optimized in compiled languages, such as SLiM (Messer 2013; Haller and Messer 2017; Haller and Messer 2019). However, for users whose scenarios are well served by the design and affordances of Geonomics, what is sacrificed in runtime will be made up for in flexibility, customizability, and ease of use. With a reasonably powerful computer and for moderately sized models, most users should not find runtime or memory a major limitation. Indeed, our first two example applications were run on a laptop computer with 8 GB of RAM and an Intel^®^ Core™ i5-8250U 3.4 GHz quad-core processor. Each run took an average of 271 s (about 0.27 s per time step) for the IBD-IBE model and 144 s (roughly 0.14 s per time step) for the simultaneous selection model. Because the polygenic adaptation example has much higher complexity, approximating the high population density of a small vertebrate, we ran it on a regular-memory node of a computing cluster (the *savio3* partition of UC Berkeley’s Savio system) with 96 GB RAM and 2.1 GHz Skylake processors in order to handle the larger memory requirement. This model took considerably longer to run (approximately 7.35 h, running at about 32 s per time step after time for upfront computation of a series of changing movement surfaces) and had a peak memory usage of 25.433 GB, but even highly complex scenarios like this remain tractable on reasonable research timelines.

**Fig. 6.**
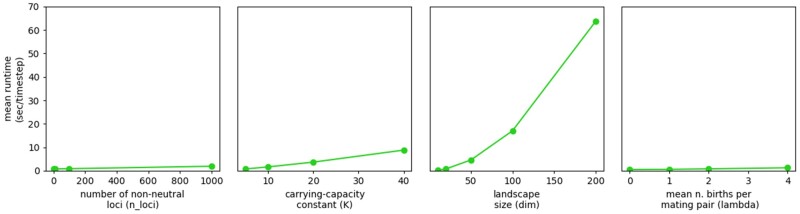
Average runtime as a function of four major parameters: the number of nonneutral loci (*n_loci*), the carrying-capacity constant (*K*) that determines mean population size, landscape size (*dim*), and the λ parameter of the Poisson distribution from which the number of offspring in each mating event is drawn (*lambda*). Runtime increases with landscape dimension both because of functions whose runtimes scale with the landscape size directly and because of functions whose runtime scales with total population size. The difference between the lines for *K* and *dim* can be taken as an indication of the runtime cost of landscape dimension above and beyond population size effects, which predominates because it is superlinear.

Given the complexity of Geonomics and the number of parameters a user can modify, numerous parameters and parameter combinations can influence a model’s average runtime. We provide a basic runtime analysis (fig. 6), run on the same 8 Gb, quad-core laptop as the examples above. This analysis highlights some basic parameters that are likely to influence a model’s average runtime per time step, including the mean population size (as determined by an array of local carrying capacities), the number of offspring per mating event, the size of the landscape, and the number of nonneutral loci in the genome. The effect of landscape size predominates, as runtime scales superlinearly with this parameter. The number of nonneutral loci actually has only slight effects on total runtime, and because neutral genetic data are stored in a set of tables rather than redundantly for each individual (Kelleher et al. 2018) runtime is even less sensitive to the number of neutral loci in the genome (although recombination rate does impact runtime and memory usage), meaning that Geonomics can efficiently simulate genome-scale data sets if provided adequate memory to store the set of recombination pathways that is calculated at the outset. Finally, after the upfront cost of computing recombination pathways and movement surfaces, runtime scales roughly linearly with the number of time steps, barring large demographic changes. That means that the moderately complex scenario in our simultaneous selection example could complete 1,000,000 time steps in approximately 40 h, and even the highly complex scenario in our polygenic adaptation example with more than 200,000 individuals could run through 10,000 generations in about 3.7 days. Hence, Geonomics could even prove useful for research at deeper timescales, for example in phylogeography or geogenomics (Baker et al. 2014).

### Caveats

Geonomics uses two unconventional approximations to make complex models tractable within reasonable compute time in an interpreted language. The first is the approximation used to model heterogeneous recombination. Enacting recombination between all neighboring loci each time a gamete is produced would require an extremely large and time-consuming number of random draws. To avoid this, when a model is first created, Geonomics generates and saves, as binary arrays, a large collection of recombination “paths.” The number of paths used is set by the user, and directly determines the minimum recombination rate difference that can be modeled. Each path is just a genome-length array that switches between 0 and 1 at each interlocus position where a recombination event should occur. The path can then quickly be used to subset an individual’s genome, producing a gamete. As a model runs and gametes are continually produced, these paths are repeatedly shuffled and drawn through, like a deck of cards during multiple rounds of a game. This approach, which we have validated using msprime (see Recombination Test, Supplementary Material online), can lead to memory limitations for models with a large number of paths and a long genome, because the data structure containing the paths is essentially a 2D binary array whose size is the product of these values and because more recombination events require that more trees are recorded in the spatial pedigree. To avoid these problems, for genomic architectures with homogeneous recombination rates, Geonomics provides the option to use an alternative recombination mechanism that simulates recombination on the fly for each new gamete but does so at a cost of increased average runtime per time step.

The second is the approximation used to model the circular distributions from which movement directions are drawn. Conceptually, a movement or dispersal surface is an x×y array of Von Mises distributions. In practice, each distribution on that surface is represented by a column of angular directions (an “approximation column”) drawn, at the time the model is built, from the true, continuous distribution. During a model run, to draw a movement direction from a cell, a random value is sampled from that cell’s approximation column. This increases computational efficiency by avoiding large numbers of calls to random number generators during runtime. The accuracy of these approximation columns is a function of their length, which is set by the user. This length will usually not be so constrained that it significantly impacts the accuracy of the approximation, but such a constraint could arise if the movement or dispersal surface undergoes environmental change. In this case, the movement surfaces corresponding to each step of the change event will be generated and stored when the model is first created, and the series of arrays produced could exhaust memory if the landscape is very large and has many environmental change steps. A solution to this problem would feature some combination of decreasing the temporal resolution of the environmental change event, decreasing the landscape size, or decreasing the approximation column length. In all cases, users may check the accuracy of modeled movement by using built-in functions that visualize the composition and behavior of movement and dispersal surfaces.

## Materials and Methods

We performed all simulations using the Geonomics Python package (see New Approaches) as described in the Results section and Supplementary Material. The simplest way to get started with Geonomics is to install it via pip. Geonomics uses common, well-established Python packages as required dependencies—Numpy (Harris et al. 2020), Matplotlib (Hunter 2007), Pandas (McKinney et al. 2010), Shapely (Gillies et al. 2007), Scipy (Virtanen et al. 2020), Scikit-learn (Pedregosa et al. 2011), Statsmodels (Seabold and Perktold 2010), Rasterio (Gillies et al. 2019), Bitarray (Schnell et al. 2021), msprime (Kelleher et al. 2016), and tskit (Kelleher et al. 2018)—and offers optional integration of neutral landscape models through the NLMpy package (Etherington et al. 2015). The source code is publicly available on GitHub (https://github.com/drewhart/geonomics), where it is actively maintained and developed.

## Conclusions

Geonomics is a Python package designed to make building and running complex landscape genomic models quick and simple. At the same time, it provides a flexible scripting framework that allows advanced users to customize and extend its functionality. We believe Geonomics will prove highly useful for theoretical, empirical, methodological, and applied research in population and landscape genomics, molecular ecology, global change biology, and conservation.

## Supplementary Material

Supplementary data are available at *Molecular Biology and Evolution* online.

## Supplementary Material

msab175_Supplementary_Data
