# Corrigendum to: Geonomics: Forward-Time, Spatially Explicit, and Arbitrarily Complex Landscape Genomic Simulations

**DOI:** 10.1093/molbev/msab250

**Published:** 2021-09-14

**Authors:** Drew E Terasaki Hart, Anusha P Bishop, Ian J Wang

*Molecular Biology and Evolution*, msab175, https://doi.org/10.1093/molbev/msab175

In the originally published version of this manuscript, there were several errors in the second paragraph of the Introduction. The end of the paragraph should read:

“To our knowledge, SLiM (Messer 2013; Haller and Messer 2017; Haller and Messer 2019) is the only package currently capable of simulating scenarios that are sufficiently complex, both genomically and geospatially, to model population genomic patterns emerging under dynamic evolutionary processes (according to a search of the National Cancer Institute’s Genetic Simulation Resources website; Peng et al. 2013), and its extreme generalizability and complexity allow it to be used for landscape genomics simulation. Furthermore, many species are distributed continuously in space, and examining continuous fields of genetic variation can require distinct methods and assumptions (Bradburd and Ralph 2019), yet most population genomic simulation packages, aside from SLiM, are population-based. Such software requires individuals to be assigned to discrete subpopulations, which can at best be arranged on a high-resolution, regular grid in order to approximate continuously distributed populations.”

This has been corrected.

