# Supplementary material for: Geonomics: Forward-Time, Spatially Explicit, and Arbitrarily Complex Landscape Genomic Simulations
Source: Mol Biol Evol. 2021 Jun 12;38(10):4634–46. doi: 10.1093/molbev/msab175 (PMC8476160; doi:10.1093/molbev/msab175)

# Supplemental Material

Geonomics: forward-time, spatially explicit, and arbitrarily complex landscape genomic simulations

*Drew E. Terasaki Hart, Anusha P. Bishop, and Ian J. Wang*

## Validation Tests

## Full details and reproducible code and parameter files for each of our validation tests are available from the ‘/tests/validation/’ subdirectory of the source code. We discuss the key details and results of these tests below.

## Wright-Fisher test: genetic drift

The Wright-Fisher model of genetic drift models a fixed-size haploid population that turns over completely at each timestep (i.e. generation). The population can have any number of independent, biallelic genetic loci. For each locus, each generation’s allele frequency is chosen as a binomial random variable, with the number of trials equal to the population size and the probability of success (i.e. of drawing the ‘1’ allele) equal to the previous generation’s ‘1’-allele frequency. The mean persistence time for an allele (i.e. the expected number of generations for which a locus remains segregating) is: $t^{‾}(p)=-4N[(1-p)ln(1-p)+p ln(p)]$ (S1),

where $2N$ is the number of alleles in the population (such that $N$can represent the diploid population size) and $p$is the frequency of an allele at the locus (Fisher, 1923; Hartl and Clark, 2007; Wright, Sewall, 1930).

The Wright-Fisher model is much simpler than the sorts of models for which Geonomics is designed (as are all of the following validation tests)—it is aspatial, panmictic, features fixed population sizes, and models only neutral loci. Thus, we parameterized Geonomics so as to approximate the model as closely as possible. To emulate aspatiality and panmixia, we used a population on a homogeneous landscape, using isotropic movement, with movement and dispersal distributions that broadly encompass the diagonal width of the landscape, and with no mating radius imposed (to allow panmixia) instead of local mating (i.e. with the mating radius set to ‘None’). To enforce complete generational turnover, we set the maximum-age parameter to 1 (i.e. 1 timestep). While Geonomics does not maintain constant population size, we maintained the carrying-capacity raster at a constant, uniform value, thus maintaining a stationary mean population size. We simulated 250 independent neutral loci (by setting all inter-locus recombination rates to 0.5), with starting ‘1’-allele frequencies of 0.5 (although the actual starting frequencies vary slightly around this value because of sampling error when all individuals’ genotypes are drawn binomially).

We ran the Wright-Fisher approximation test for three values of the carrying-capacity raster (i.e. three values of ‘K_factor’), hence for three mean population sizes (708, 1564, and 2440 individuals). For each mean population size (calculated as the harmonic mean, to account for stochastic fluctuations around the carrying capacity), we compared mean persistence time to that expected by theory, according to equation [S](#bookmark=id.3rdcrjn)1. Figures S1 and S2 show that the results are a close match to the theoretical expectations.

## Bottleneck test: population dynamics

Because drift is a stronger evolutionary force in smaller populations, drift accelerates in shrinking populations. If a population undergoes a bottleneck event, the overall effect of drift on the population during that time is expected to be larger than what a constant-size population of equivalent starting size would experience during that time. Thus, mean fixation time should decrease in a bottlenecked population relative to one of constant size.

As with the Wright-Fisher model, to test the effectiveness of Geonomics for modeling a population bottleneck we used a homogeneous landscape with broad distributions for movement and dispersal and without a mating radius in order to emulate aspatiality and panmixia. To simulate a bottleneck event, we created a custom change event in which the population’s carrying-capacity raster is reduced to 30% of its initial value for 50 timesteps (from the 200th to 250th), then returned to its initial value for the remainder of the simulation (through the 300th timestep). These simulations produced a clear signal of accelerated drift during the bottleneck event, with the mean rate of allele-frequency change, calculated in 15-timestep sliding windows, nearly tripling during the period of the bottleneck (Figure S3).

## Stepping stone test: population subdivision and genetic differentiation

The stepping-stone model, or one-dimensional island model, is a spatially implicit model. It models a series of subpopulations arranged along a straight line, with migration between all neighboring pairs. As a combined result of divergence by drift and homogenization by effective migration, subpopulations are expected to reach a stationary level of genetic differentiation: migration-drift equilibrium. Theory provides the expected pairwise genetic differentiation between a pair of subpopulations at equilibrium as:

$F_{ST}=\frac{1}{1+4Nm}$ (S2),

where $N$ is the population size and $m$ is the per-generation migration rate, such that $Nm$ can be interpreted as the per-generation number of migrant individuals (Hartl and Clark, 2007).

To approximate the stepping-stone model, we created a Landscape Layer with a diagonal of six equally spaced, equal-sized islands (1.0-valued cells) embedded in a ‘sea’ of 0.0-valued cells. We used this layer as the carrying-capacity raster (Figure S4, left). We parameterized dispersal to be very near to parents’ midpoints, movement distance to be strongly right-skewed, such that the long-distance movement events leading to migration are uncommon, and the mating radius to a value that makes island populations effectively panmictic but that prohibits mating between individuals on separate islands. Genomes contained 100 neutral loci, and we ran the simulation for 5000 timesteps.

Because Geonomics does not model discrete populations, it does not stipulate migration rates between discrete locations on the landscape. Thus, we manually tracked the number of migration events during each timestep for all possible directional migration events (i.e. for all permutations of island pairs), then used that data to calculate all mean migration rates. With those values, we solved equation S2, then compared the resulting $F_{ST}$expectations to the observed values (calculated from the simulated data using two common methods; Figure S4, right). We also used Discriminant Analysis of Principal Components (DAPC), performed in the R package *adegenet* (Jombart et al. 2008), to visualize population structure.

The results demonstrate that the model approached migration-drift equilibrium, as expected by theory (Figure S5), with all island populations reaching dynamic equilibria around the same mean size. Estimated migration rates and $F_{ST}$values qualitatively match theoretical expectations: mean migration rate drops off precipitously at greater than one step-distance apart, and genetic differentiation increases to approximate saturation. Values of $F_{ST}$consistently undershoot the values expected based on estimated migration rates, however, because subpopulations have yet to approach fixation at most loci (which is the expectation implied by expected $F_{ST}$values close to 1). DAPC demonstrated that the simulation generated the expected population structure of six distinct clusters, one per island (Figure S6).

## Contrasting-habitat test: divergent selection

In a population divided between two, divergent selective environments, if there is standing genetic variation for a biallelic locus controlling the trait adapting to those environments then theory predicts that the two subpopulations will diverge at that locus as each moves toward its respective adaptive peak. The rate at which divergence should occur depends on the relative strengths of two opposing evolutionary forces: natural selection, which causes divergence, and gene flow, which causes homogenization. The rate of allele frequency change in either subpopulation at timestep t is expressed as:

$\delta q=\frac{-spq[q+h(p-q)]}{1-sq(2hp+q)}+m_{i}q^{*}-m_{o}q$ (S3),

where $p$and $q$are the frequencies of the beneficial and deleterious alleles in the local subpopulation, $s$is the selection coefficient against the homozygous recessive phenotype, $h$is the degree of dominance of the recessive allele, *m_i_* and *m_o_* are the migration rates into and out of the subpopulation being analyzed, and $q^{*}$is the frequency of the locally deleterious allele in the alternative subpopulation where it is beneficial (Hartl and Clark, 2007).

This model, like the stepping-stone model, is spatially implicit. To approximate this, we created a landscape with two layers. The first was divided into two equal-sized halves, one valued at 0.0, the other at 1.0; this layer was used as the layer driving natural selection. The second was valued uniformly at 1.0; this was used as the carrying-capacity raster (thus setting uniform population density across the landscape and determining, in sum, the overall carrying capacity of the landscape). We created one monogenic trait whose position was randomly chosen within a genomic architecture of 100 unlinked loci. We ran the model for 1000 timesteps for each of three values of the parameter ɸ (identical to *s* in equation S3): 0.1, 0.05, and 0.01. Given that Geonomics does not directly define a migration rate parameter, we tracked the number of migration events (i.e. individuals crossing the landscape’s horizontal midline) during each timestep, then used that data to solve equation S3.

Results depict clear local adaptation to each of the two halves of the landscape, with spillover of opposite phenotypes and resulting heterozygote births occurring along the border between the two habitats (Figure S7). Allele trajectories in each half of the environment follow qualitatively the increasing and saturating trajectories expected by theory, but reach consistently more divergent allele frequencies than expected based on the theoretical calculation (Figure S8). However, these results are an easily understandable artefact of estimating a spatially implicit, population-based model using a spatially explicit, individual-based one—our method of calculating migration rates includes all individuals who cross the habitat boundary, including the large number who only barely cross and who may even quickly migrate back, such that expected allele-frequency trajectories are based on an overestimation of true gene flow and thus serve as lower bounds on the real trajectories. As further validation, a plot of the mean difference between each individual’s phenotypic and environmental values shows a strong decline over model time, with the rate and level of decline increasing as a function of increasing strength of selection (Figure S9). Moreover, logistic regressions show no significant relationships between phenotypic and environmental values at the outset (pseudo-R^2^s ≈ 0.0, p-values > 0.1) but show highly significant relationships at the ends of the simulations (p < 0.0001 for all values of ɸ), with the amounts of variation explained increasing as a function of selection strength (pseudo-R^2^ = 0.327 for ɸ = 0.01, 0.376 for ɸ = 0.05, and 0.406 for ɸ = 0.1).

## Cline test: local adaptation

In a clinal model, a population adapts locally across an environmental gradient, which is characterized by the extremes of its environmental values and its steepness (i.e. the instantaneous rate of environmental change along it). Local adaptation across this gradient will generate a geographic cline in allele frequencies. The clinal pattern is only expected for loci under selection along the cline (and other loci in linkage). Unlinked loci have no long-term clinal expectation (though they could initially be swept along with the selective locus, and any number could continue to show spurious concordant clinal variation). To detect clinal adaptation, we can fit cline curves to the allele-frequency variation across the environmental gradient for all loci, with the expectation that the clines fit to adaptive loci will mirror the gradient. Numerous equations have been used to fit clines, but one of the most common is the sigmoidal *tanh* function:

$p_{x}=\frac{1}{2}(1+t[\frac{2(x-c)}{w}])$ (S4),

where $p$ is the frequency of the reference allele at position $x$ along the cline, $c$ is the centerpoint of the cline (such that $p_{x=c}=0.5$), and $w$ is the ‘width’, which is defined as $w=\frac{1}{slope}$ at point $c$ (Porter, 2013).

To implement the cline model in Geonomics, we created a landscape with two layers. The first layer was an environmental layer—a symmetrical, non-linear gradient between 0-valued and 1-valued halves (Figure S10). The second was a uniformly valued habitat-quality layer, used to set a uniform population density and thus determine the global carrying capacity. We created a monogenic trait whose locus was randomly placed within a genomic architecture of 100 independent loci. The trait had a ɸ of 0.01, with the gradient layer serving as its selective force. We ran the cline model for 1500 timesteps, then used a numerical optimization function (in Python’s *scipy* package; Jones *et al.*, 2001) to fit equation S4 for all loci. We plotted all fitted clines on top of the first landscape layer, with the cline for the single selective locus highlighted. The selective locus consistently and clearly stands out as the only locus with a cline matching the expectation of a monotonic pattern mirroring the environmental gradient and spanning nearly the full range of phenotypic values (Figure S11).

Results clearly show a pattern of clinal adaptation across the landscape—despite isolated patches of maladaptive genotypes potentially resulting from occasional long-distance migration events—with a zone of admixture and phenotypic spillover surrounding the cline’s center (Figure S10). In a Bonferroni-corrected family of locus-wise logistic regression models of environmental value on genotype, the selective locus consistently stands out as the most significant (p-values of roughly 3x10^-100^). Furthermore, a plot of the mean difference between phenotypic and environmental values shows a strong decline over model time (Figure S12), and logistic regressions show no significant relationship between phenotypic and environmental values at the outset (pseudo-R^2^ = 0, p-value = 0.370) but a significant relationship at the end of the simulation (pseudo-R^2^ = 0.345, p-value < 0.0001).

## Selective sweep test: genetic hitchhiking

Genomic architecture and linkage add important complexity to models of molecular evolution. The most basic model of selection with linkage is that of a selective sweep: a beneficial mutation occurs in a population, falling on a random genomic background, then rises in frequency because of its selective advantage until it becomes fixed, pulling up the frequency of the surrounding haplotype block in the process. The haplotype block is, nevertheless, subject to recombination, which gradually erodes it symmetrically around the beneficial mutation. Thus, the selective-sweep model predicts that once a beneficial mutation occurs —as long as it is not lost early on by chance— it and the haplotype block around it will rise in frequency, the mutation will eventually fix, potentially with some core block around it, and the rest of the block will erode over time. The haplotype block should be clearly visible in genomic data, where it will manifest as a genomic region of reduced diversity and heterozygosity centered on the mutation.

To implement the selective sweep model in Geonomics, we again created a model approximating an aspatial, panmictic population (see Wright-Fisher test for details). We created a single, monogenic trait with a ɸ of 0.1. The trait’s locus was manually set to position 500, such that it was at the center of the 1001-locus genome. The genome had a homogeneous recombination rate of 0.001 between all neighboring loci. We manually set the starting ’1’-allele frequency at this locus to 0.0 but set the trait to be selected upon by a uniform layer of 1 values, such that all individuals began the model equally unfit (i.e. with a fitness value of $1-\phi=0.9$). After burn-in, we iteratively chose a random individual, introduced a ‘1’-mutation in its genome at locus 50, ran the model for 50 timesteps, and checked whether the ‘1’ allele had reached a frequency greater than 0.05 by that time. We iterated until that check was passed, at which point we declared the mutant allele ‘established’ and continued to run the model until 2500 timesteps after the novel mutation reached fixation. At three timepoints during that model we calculated and recorded genome-wide nucleotide diversity using a sliding-window approach.

We found that Geonomics successfully and realistically simulated the behavior of a selective sweep. The first adaptive mutant that was not immediately lost by drift rose rapidly in frequency, then fixed. The population’s mean fitness increased quickly from 0.9 (the universal fitness value before the mutation was introduced) to 1.00 (the universal fitness value after the sweep was complete; Figure S14). The linkage block around the selected locus became a region of depressed nucleotide diversity (Figure S13, top row) and heightened linkage (Figure S13, bottom row) ⁠— the classic signature of a selective sweep.

## Recombination test

To provide additional validation of Geonomics’ recombination model, we compared the effective recombination rates observed in a Geonomics model to those produced by an msprime simulation using the same recombination map. We produced a recombination map by assigning 999 random, interlocus recombination rates to a 1000-length simulated genome. We drew the rates by taking the first 999 values ≤ 0.5 from a random vector drawn from the distribution ~Beta(0.4, 1.3), producing a left-skewed distribution that nonetheless sampled the full range of physical linkage values. We ran an msprime model using the msprime.RecombinationMap object created from those values and also ran a Geonomics model using those values as the recombination-rate column in a Geonomics custom genomic architecture file. We then plotted the true recombination rates and the observed breakpoint densities from both models, binned within even-width genomic windows. The results show that Geonomics’ observed breakpoint densities recapitulate the true recombination rates just as closely as do those of msprime (Figure S15).

## Applications

*Example 3: Polygenic adaptation to climate change in the Yosemite region*

To build this simulation, we first generated a template Geonomics parameters file (using the Geonomics function ‘gnx.make_parameters_file(…)’), then edited it to best emulate our empirical study system (see Code Sample S1). This created a parameters file for a simulation with: (1) three empirical layers mean temperature, precipitation, and habitat suitability), two of which (temperature and habitat suitability) have environmental change events; (2) one species, with one trait adapted to mean temperature; and (3) a data collection design. We set life-history parameters to reasonable approximations of *S. graciosus* biology, based on available literature. We set spatial parameters based on the relationship between the resolution of the environmental rasters and the characteristic scales of *S. graciosus* key life-history traits. The resolution of the rasters is 0.00833°, which at latitude 38° is equal to about 730.984 m in the east-west direction and 927.296 m in the north-south direction, giving each cell a total area of roughly 6.78 × 10^5^ m^2^ (67.8 hectares). Using a population density of 208 individuals per hectare (Tinkle, 1973) and the rough estimate that about 10% of the land area covered by our study contains the open habitat favored by *S. graciosus* rather than the more closed habitat favored by the congener *S. occidentalis*, with whom it experiences a large degree of competitive exclusion (Rose 1976), we chose a per-cell carrying capacity (parameter ‘K_factor’) of 67.8 hectares/cell × 208 individuals/hectare × 0.1 proportion of habitat suitable ≈ 1410 individuals (which we then further multiplied by 0.1 for computational tractability). We set the reproductive age to 2 years (Tinkle, 1973; Tinkle *et al.*, 1993). We left the sex ratio at unity, given controversy in the literature about whether or not it skewed toward females because of lower male survival rates (Tinkle, 1973; but see Tinkle *et al.,* 1993). We set the number of births per individual to be a Poisson random variable with lambda = 4.464 individuals/clutch × 2 clutches/year × 0.16 survival rate = 1.428, based on an average clutch size of 4.464 across surveyed California populations (Tinkle *et al.* 1993), an average of 2 clutches per year (Tinkle, 1973; Tinkle *et al.,* 1993), and an average rate of survival to the first year of 0.16 (Ruth, 1978). We estimated the mean interannual movement distance as 12.457 m (expressed as 0.01704 cell widths), based on an average of all recorded interannual movement events in Stebbins’ (1948) study of *S. graciosus* home ranges. We used this as an order-of-magnitude estimate for movement, but increased parameters slightly above this value in order to pair the reduced population density we chose for purposes of computational tractability. Thus, we set a mating radius value of 0.5 cell widths, and parameterized movement and dispersal as ‘~Lognormal(7x10^-5^, 0.3)’ and ‘~Lognormal(7.5x10^-4^, 1),’ respectively. In the absence of any known published estimates, we set the population intrinsic growth rate to 0.5. The full code to perform this analysis is available as Code Sample S2 and in the Yosemite demo script, included in the Geonomics package.

## Accessibility

For both of the two most common types of color blindness (protanopia and deuteranopia) we tested the colorblind-friendliness of all of the Matplotlib color palettes used by Geonomics as defaults. For perceptive simulation we used the script provided by Sarjak Thakkar (2018). All color palettes retain interpretability.

#

# Figure Legends

**Figure S1:** Trajectories for the frequencies of the ‘1’-alleles for 25 of the 250 simulated loci (one line per locus) in a Wright-Fisher model without mutation. We ran simulations for three mean population sizes, as determined by three fixed values of the carrying capacity (‘K_factor’) parameter, until all loci fixed.

**Figure S2:** Violin plots of mean persistence time distributions across all loci from our Wright-Fisher validation test, shown as a function of harmonic mean population size. Resulting mean persistence times (red dots) are an extremely close match to predictions calculated using Equation S1 (black, horizontal lines).

**Figure S3:** Ten randomly chosen allele frequency trajectories (top), population size (middle), and mean rate of allele frequency change (bottom; calculated for 15-timestep sliding windows) from the bottleneck validation test. We ran the simulation for 300 timesteps with a 50-timestep bottleneck.

**Figure S4:** Map of six island populations at the end of the simulation for the stepping-stone validation test (left), produced using ‘model.plot’ in Geonomics, and plot of pairwise $F_{ST}$ values and inter-island migration rates as functions of inter-island distance (right). $R^{2}$ values and p-values result from quadratic regressions of $F_{ST}$ values on inter-island distances and log-log regression of mean migration rates on inter-island distances.

**Figure S5:** Plot of $F_{ST}$ over model time for the stepping-stone validation test. Each line represents a different island pair, with colors corresponding to increasing inter-island distances (from yellow to green).

**Figure S6:** Results of discriminant analysis on principal components (DAPC) from the stepping-stone validation test, including plots of (A) the individual loadings on the first three discriminant axes, (B) individuals at the final time step color coded by population membership assignments, and (C) DAPC membership probabilities for each of the individuals. The optimal number of PCs to retain (n = 59) was determined through cross-validation using the ‘xvalDapc’ function in the adegenet R package (Jombart et al. 2008).

**Figure S7:** Map of the population after spatially divergent selection at $\phi$ = 0.10 in simulations for the divergence validation test, produced using the ‘model.plot_fitness’ function in Geonomics. Individuals are plotted on top of the selective landscape layer, which is divided into two halves. Outer circles are colored by phenotype, ranging from dark blue to dark red, representing the optimal phenotypes for each environmental background. Inner circles are color and sized by fitness, such that darker-gray, larger inner circles represent less fit individuals. Stochasticity leads to asymmetry in the structure of the hybrid zone, the nature of which varies from one iteration to the next; at the moment when this figure was produced, more blue alleles were present in the red environment than vice versa.

**Figure S8:** Observed (solid lines) versus expected (dashed lines) allele-frequency trajectories for two contrasting habitats (blue = 0.0-valued; red = 1.0-valued) resulting from divergence test simulations with three selection coefficients:$\phi$ = 0.01 (dark), $\phi$ =0.05 (medium), and$\phi$ = 0.10 (light).

**Figure S9:** Plot of the mean difference between each individual’s phenotype and environmental value plotted against time, for divergence test simulations with three different selection coefficients (‘phi’). A pattern of background matching, which is indicative of local adaptation, builds up over time. The pattern develops more quickly, and becomes more pronounced, under stronger selection regimes.

**Figure S10:** Map of the final generation from the cline test simulation on top of the selective landscape layer, with individuals colored by phenotype (outer circles) and fitness (inner circles), as in Figure S7.

**Figure S11:** Plot of allele-frequency clines (neutral loci in black, selective locus in bold yellow) against the selective landscape layer (horizontal gradient from red to blue) from the cline test.

**Figure S12:** Plot of the mean difference between each individual’s phenotype and environmental value plotted against time during the cline test simulation. A pattern of background matching builds up over time.

**Figure S13:** Results of the selective sweep validation test, including nucleotide diversity calculated in 11-locus windows across the genome (top row) and pairwise linkage (R^2^) for locus pairs plotted against genetic distance (bottom row). Genetic distance was calculated as the ‘recombination distance’ (the sum of intervening interlocus recombination rates between paired loci).

**Figure S14:** Mean fitness of the entire population, over the full run of the selective sweep test simulation.

**Figure S15:** Breakpoint densities, within 50, even-width genomic windows, as calculated from the tskit.TreeSequence results of a Geonomics simulation (red) and an equivalent msprime simulation (blue). The observed densities clearly recapitulate the true recombination rates expressed in the input recombination map (black dashed line).

**Figure S16:** Results from discriminant analysis of principal components (DAPC) of neutral-locus genotypes at the final time step from the simulations for the isolation by distance (IBD) and by environment (IBE) example, conducted using the R package *adegenet* (Jombart et al. 2008). The optimal number of PCs to retain (n = 7) was determined through cross-validation using the ‘xvalDapc’ function (Jombart et al. 2008). A plot of the individuals’ loadings on the first three discriminant axes (A), with each individual colored according to its DAPC-derived population membership assignment (C), recapitulates their spatial arrangement (B). Beyond showing general patterns of IBD, our simulated neutral genetic data clearly match the expected hierarchical population structure: distinct clusters separated by the central barrier, with the subclusters further differentiated along the environmental gradients running in opposite directions on either side.

**Figure S17:** The mean difference between individuals’ phenotypes and environmental values plotted against time, resulting from the simulations for the simultaneous selection example application. Values decrease over time for both traits, reflecting the buildup of a pattern of background matching.

**Figure S18:** Results of the simultaneous selection simulation when selection is excluded (ɸ = 0). Individuals are colored by phenotype for the trait corresponding to each layer and show no signal of background matching, as expected.

**Figure S19:** The mean difference between individuals’ phenotypes and environmental values plotted against time, during the simultaneous selection simulation, when selection is excluded (ɸ = 0). These values show no decreasing trend over time, unlike in the model including selection (Figure S16).

#

# Code Samples

1. **Code Sample S1:**
2. >>> gnx.make_parameters_file(filepath=‘yosemite_params.py’,
    layers=[{‘type’: ‘file’, ‘change’: True},
    {‘type’: ‘file’, ‘change’: True},
3. {‘type’: ‘file’, ‘change’: False}],
    species=[{‘movement’: True,
4. ‘movement_surface’: True,
    ‘genomes’: True, ‘n_traits’: 1}],
    data=True)
6. **Code Sample 2:**
7. >>> model = gnx.make_model(filepath=‘yosemite_params.py’)
8. >>> model.walk(100000, mode = ‘burn’)
9. >>> model.walk(500, mode = ‘main’)

# References

Fisher, R.A. (1923). XXI.—On the Dominance Ratio. Proc. R. Soc. Edinb. *42*, 321–341.

Hartl, D.L., and Clark, A.G. (2007). Principles of Population Genetics: Fourth Edition (Sunderland, Massachusetts: Sinauer Associates, Inc. Publishers).

Jones E, Oliphant E, Peterson P, *et al.* (2001). SciPy: Open Source Scientific Tools for Python, http://www.scipy.org/.

Porter, A. (2013). ClineFit v. 2.0, User’s Manual.

Rose, Barbara R. 1976. Habitat and prey selection of *Sceloporus occidentalis* and *Sceloporus graciosus*. Ecology. 57, 3:531-541.

Ruth, SB. 1978. A Comparison of the demography and female reproduction in sympatric western fence lizards (*Sceloporus occidentalis*) and sagebrush lizards (*Sceloporus graciosus*) on Mount Diablo, California. Ph.D dissertation: University of California Berkeley. URL: https://elibrary.ru/item.asp?id=7216038.

Stebbins, Robert C. 1948. Additional observations on home ranges and longevity in the lizard *Sceloporus graciosus*. Copeia. 1: 20-22.

Tinkle, Donald W. 1973. A population analysis of the sagebrush lizard, *Sceloporus graciosus* in Southern Utah. Copeia. 2:284-296.

Tinkle DW, Dunham AE, Congdon JD. 1993. Life history and demographic variation in the lizard *Sceloporus graciosus*: a long-term study. Ecology. 74, 8:2413-2429.

Thakkar S. 2018. Simulate-Correct-ColorBlindness. GitHub Repository: https://github.com/tsarjak/Simulate-Correct-ColorBlindness. Commit: 95edbbbecaa75e4869e0b7c12126a6965445c93c.

Wright, Sewall (1930). Evolution in Mendelian populations. Genetics *16*, 97–159.

# Figures

1. **Figure S1:**


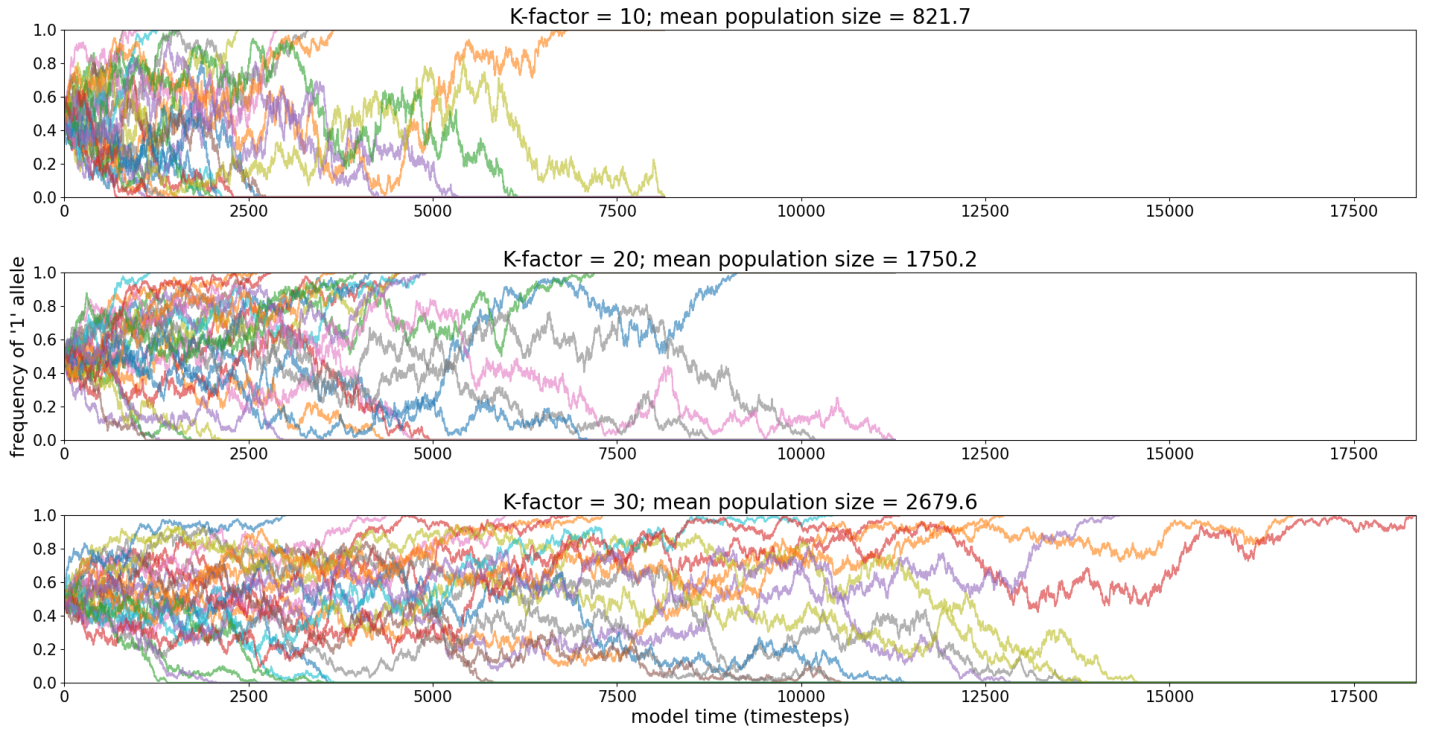


**Figure S2:**


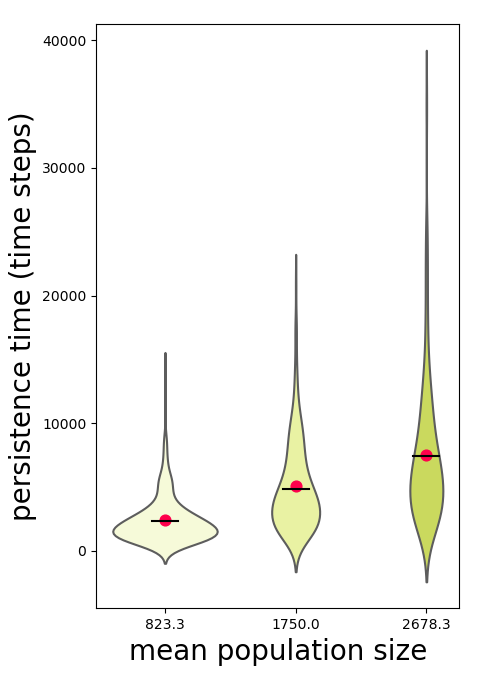


**Figure S3:**


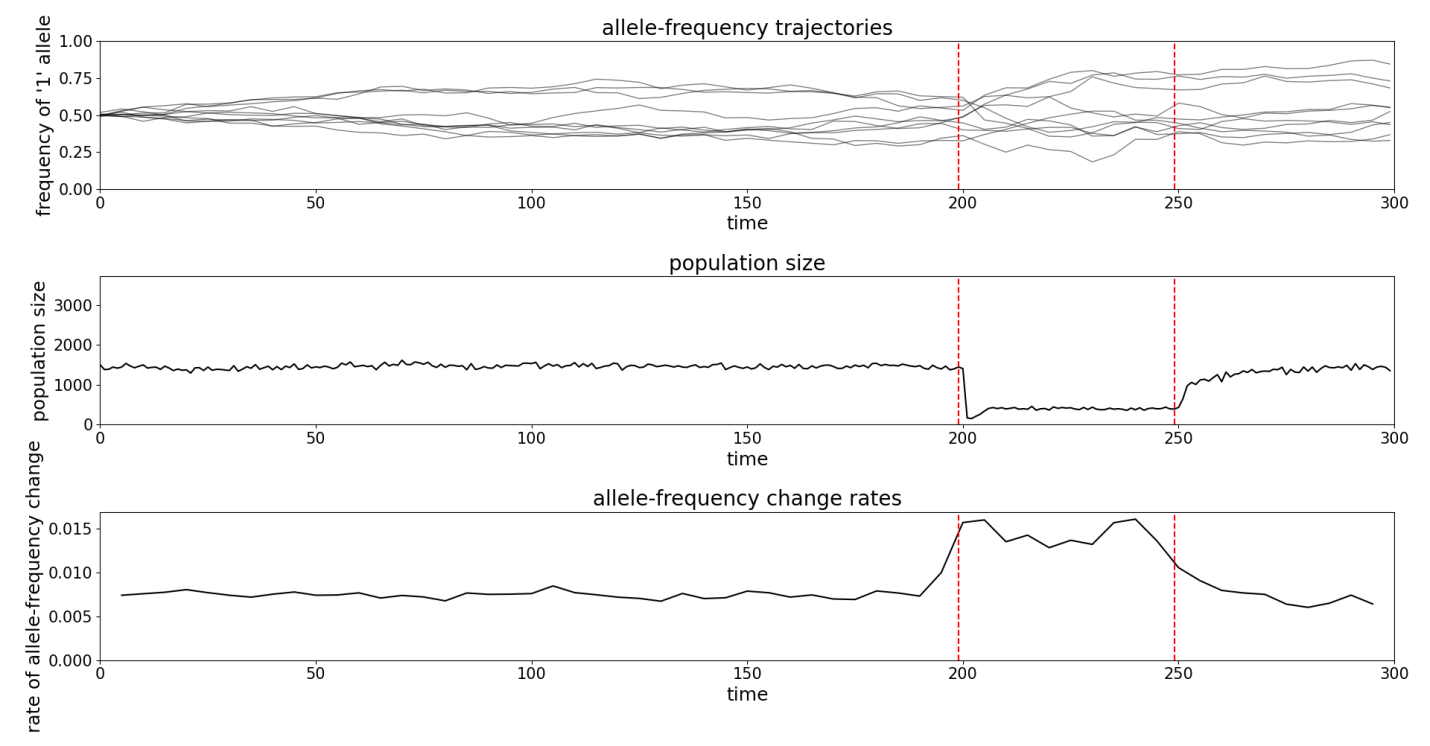


**Figure S4:**
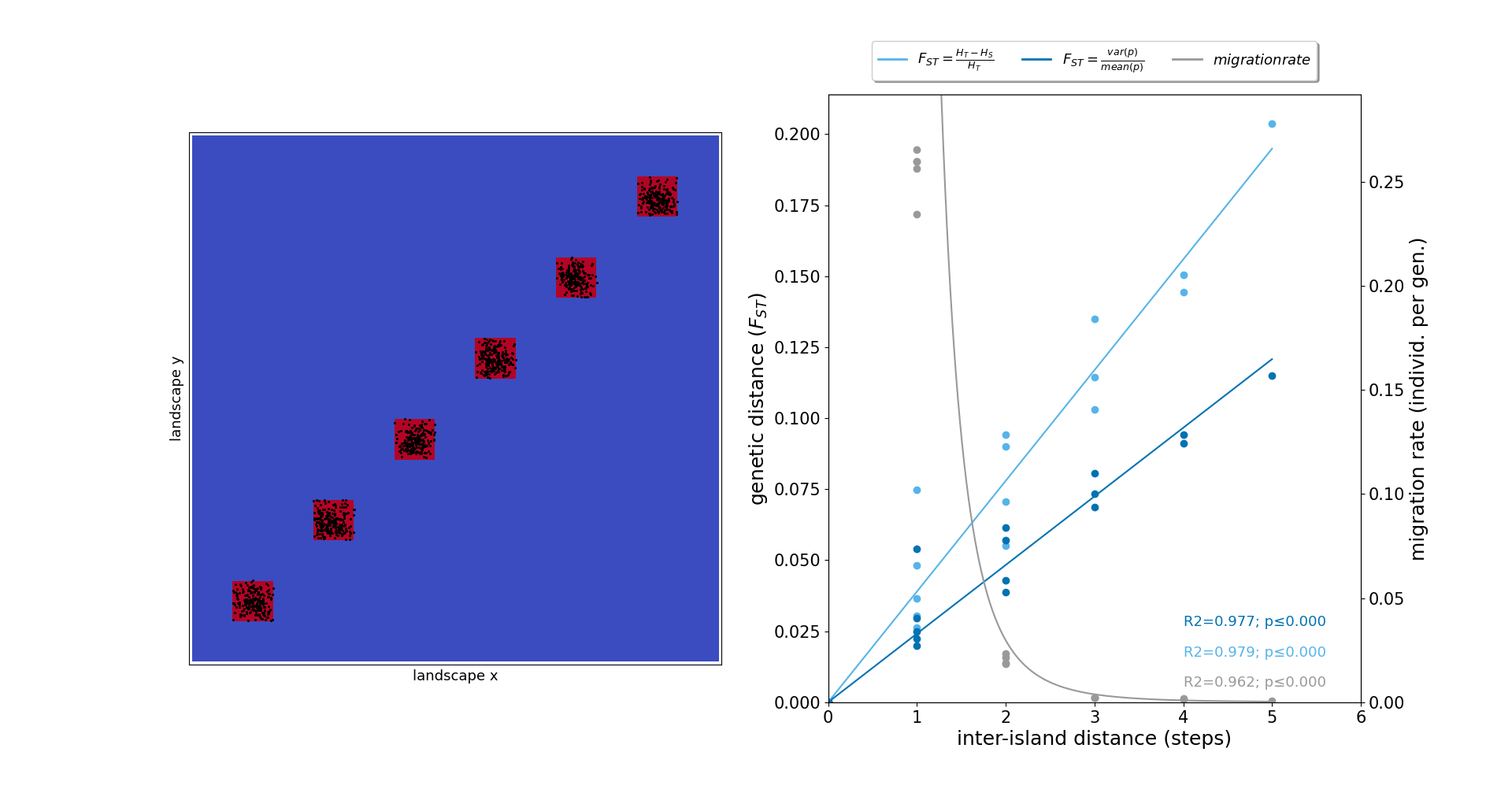


**Figure S5:**


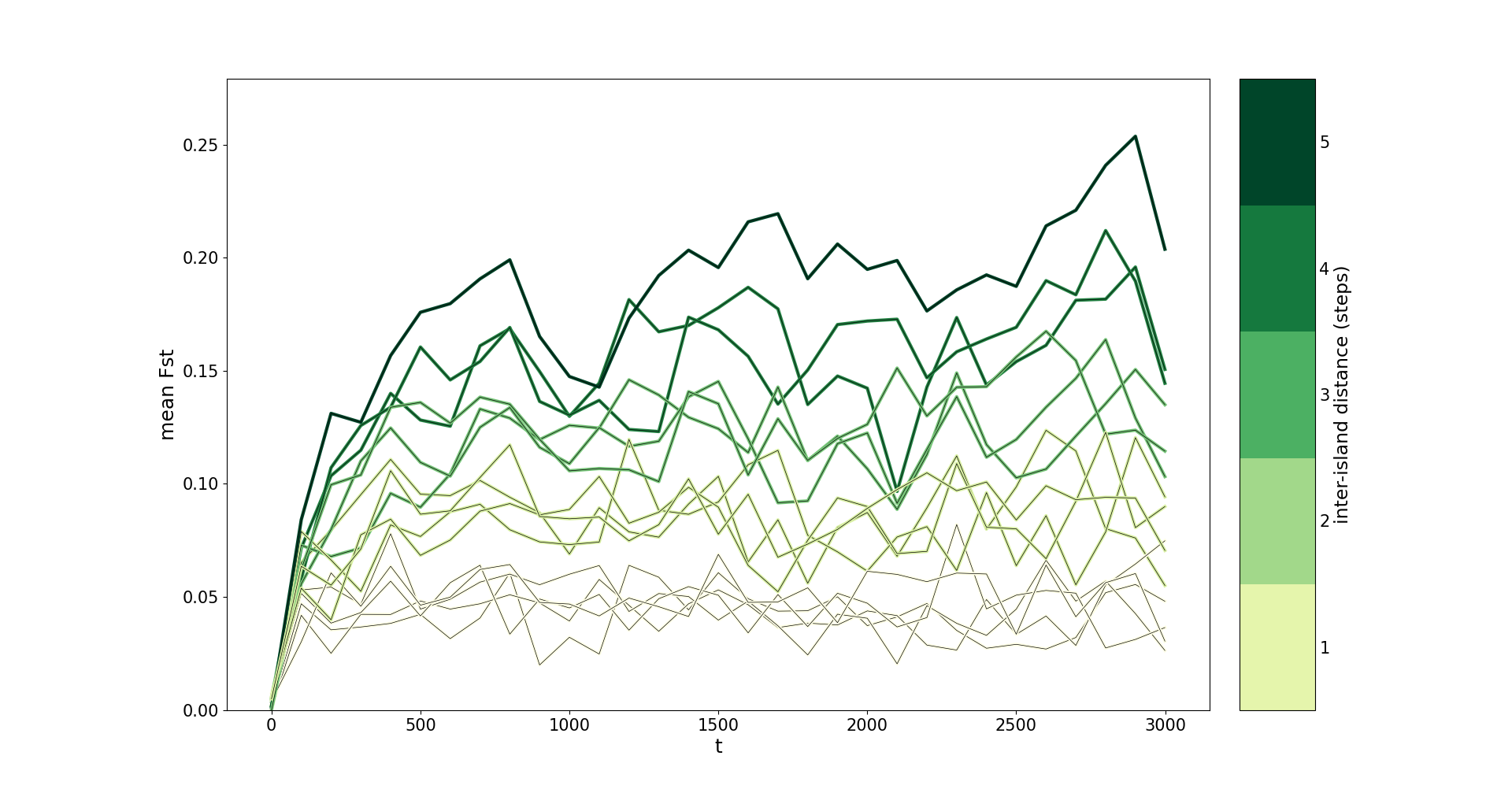


**Figure S6:**

*
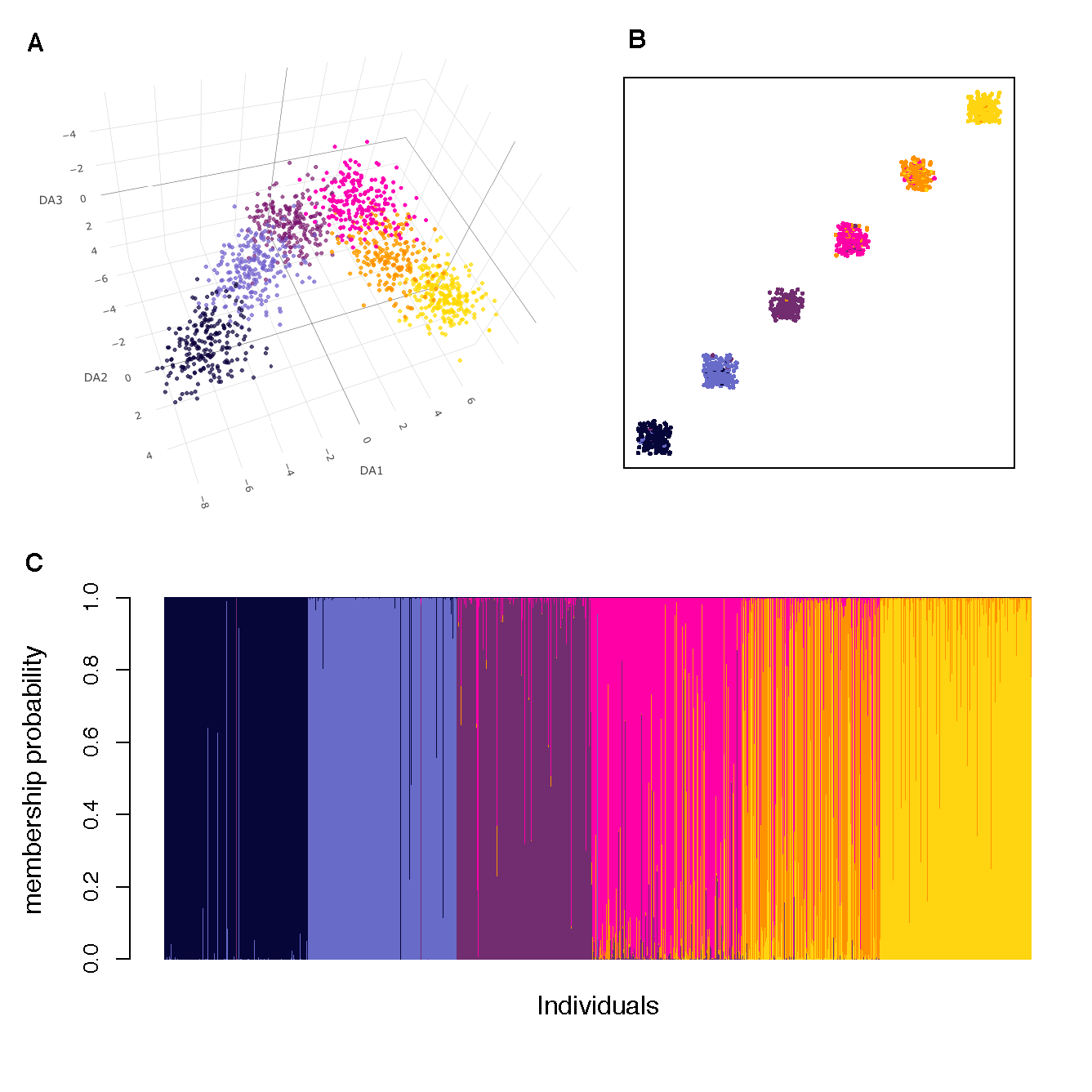
*

**Figure S7:**


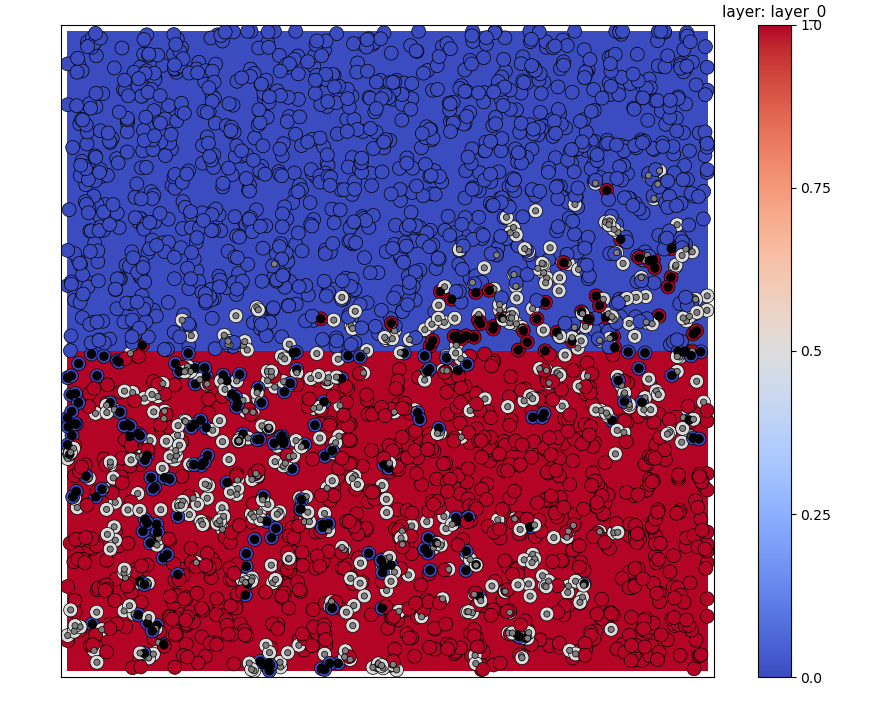


**Figure S8:**


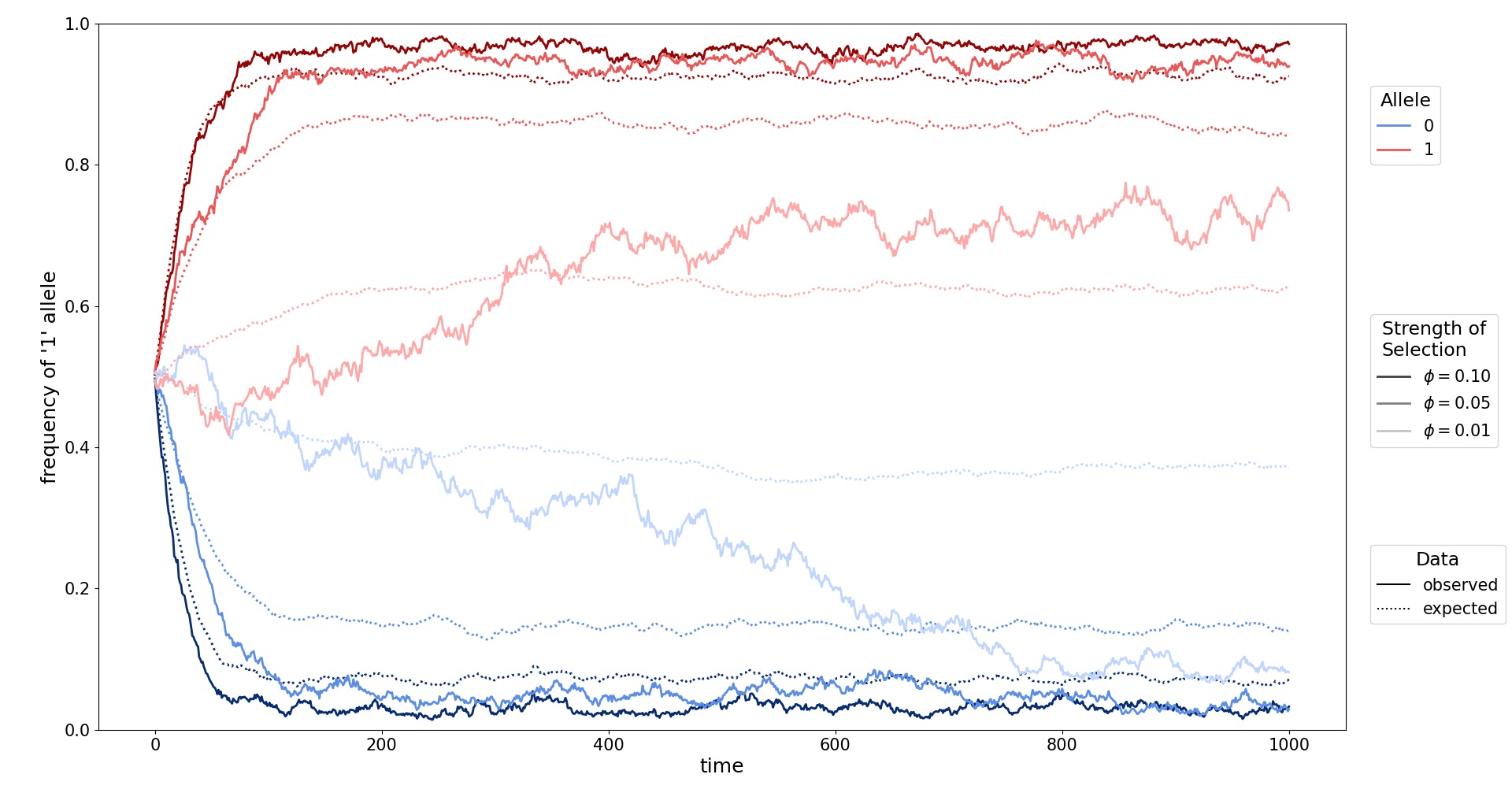


**Figure S9:**


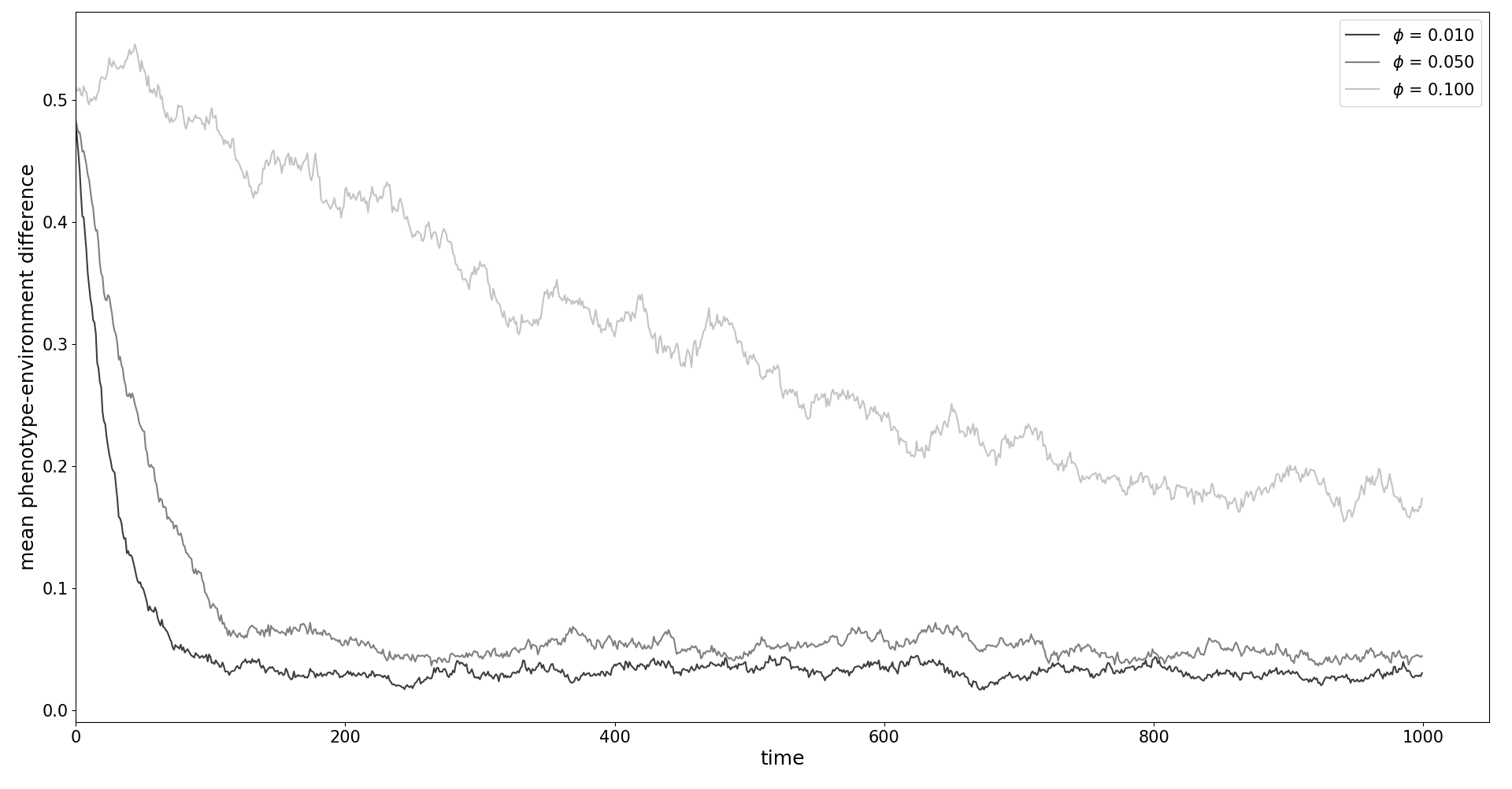


**Figure S10:**


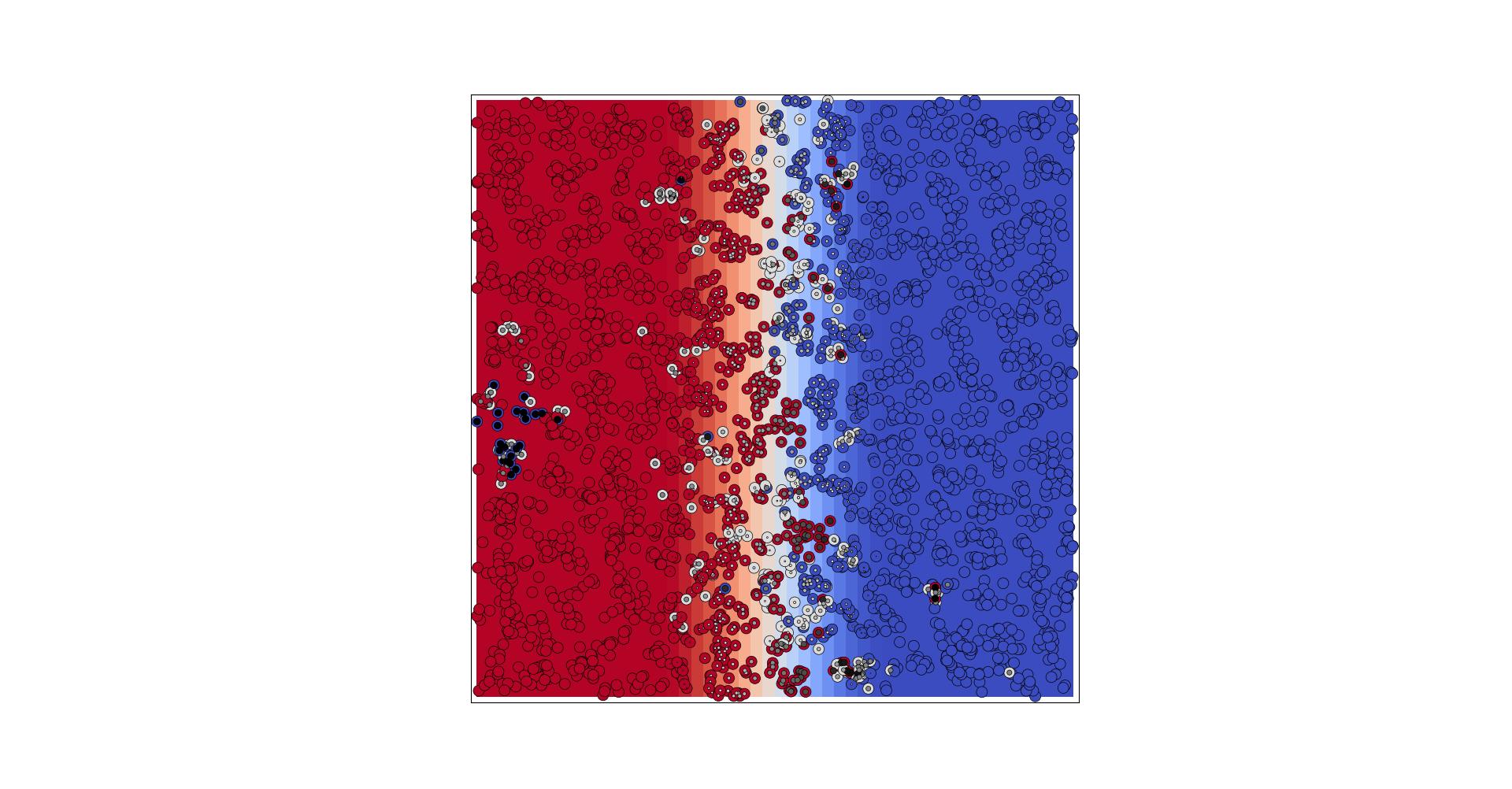


**Figure S11:**


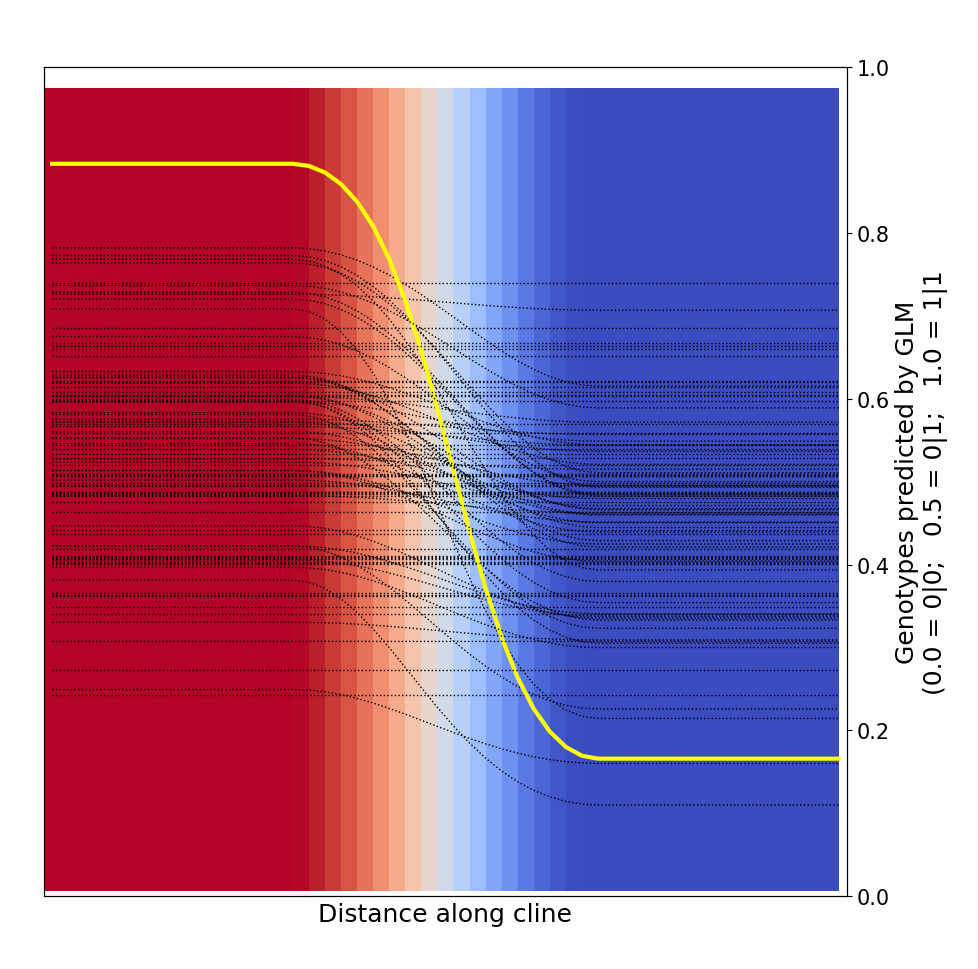


**Figure S12:***
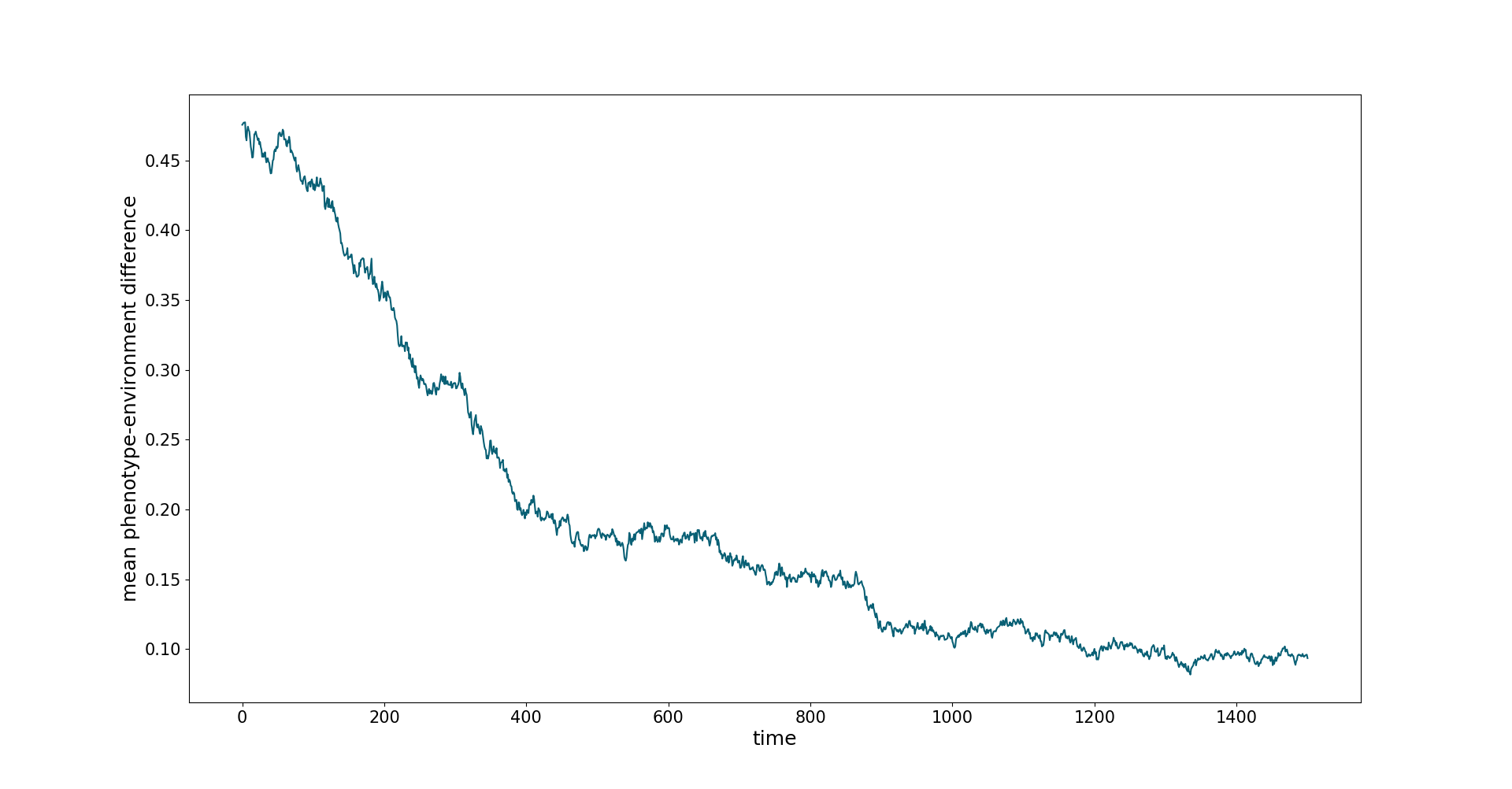
*

**Figure S13:**


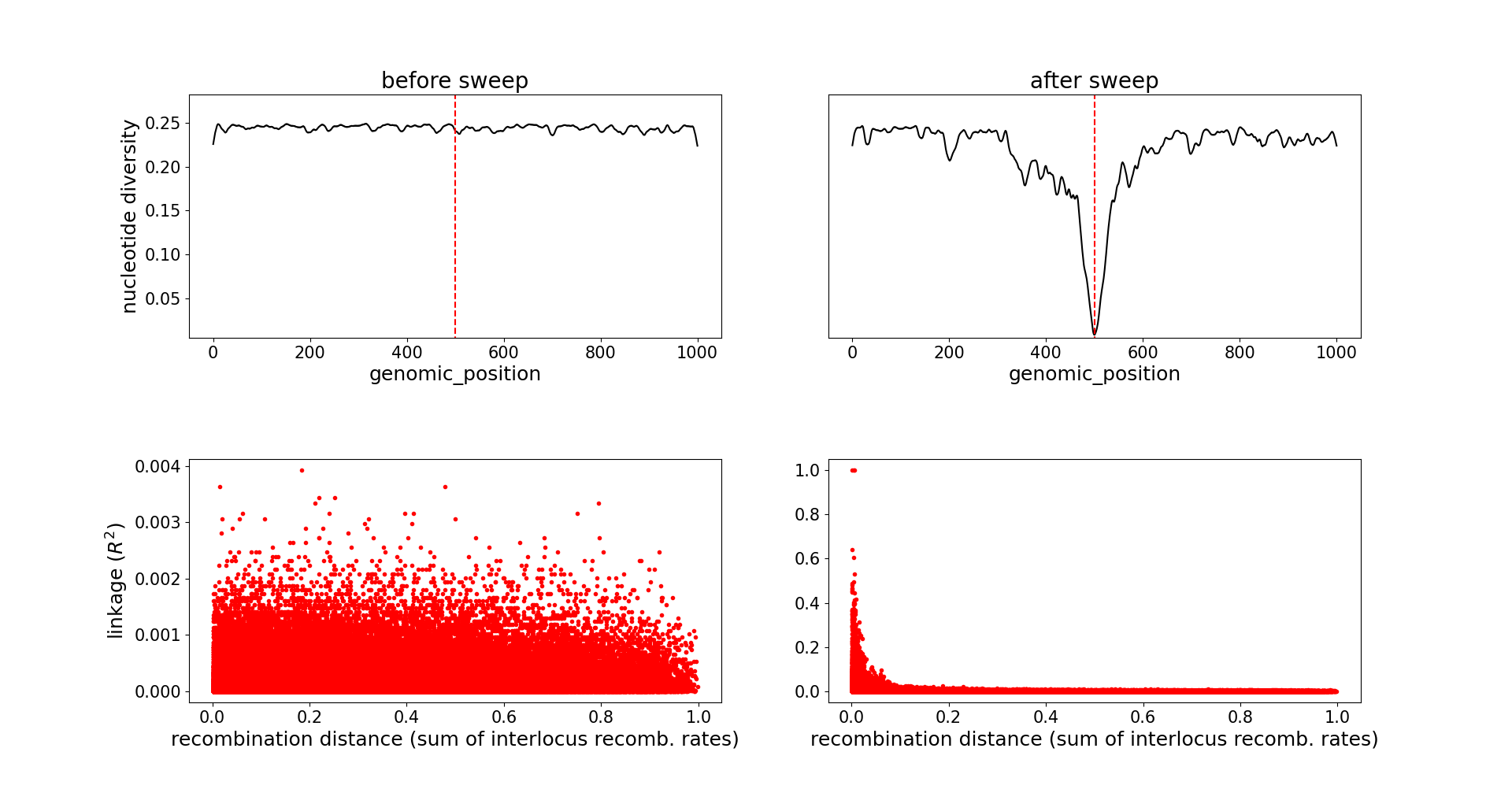


**Figure S14:**


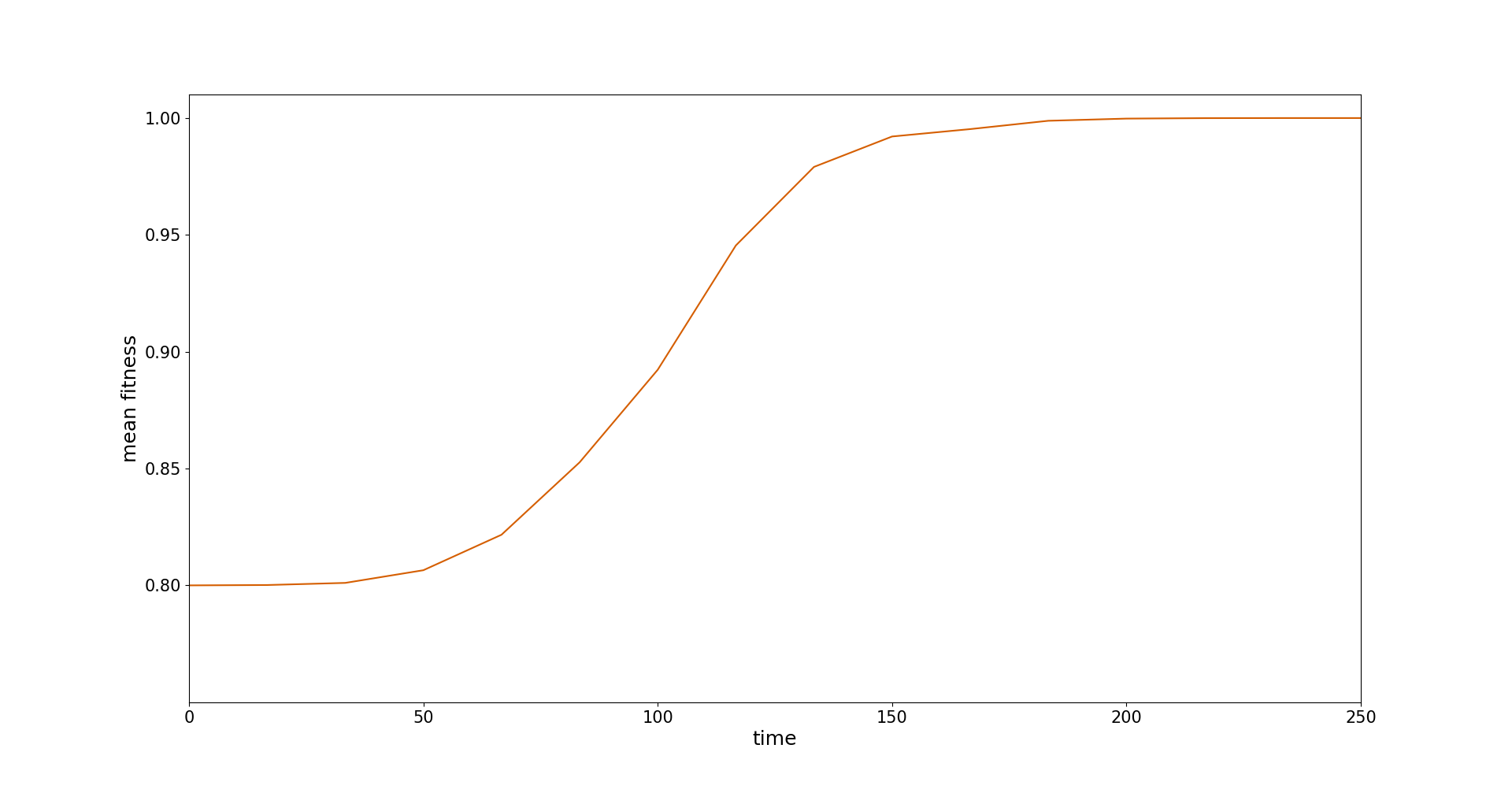


**Figure S15:**

**
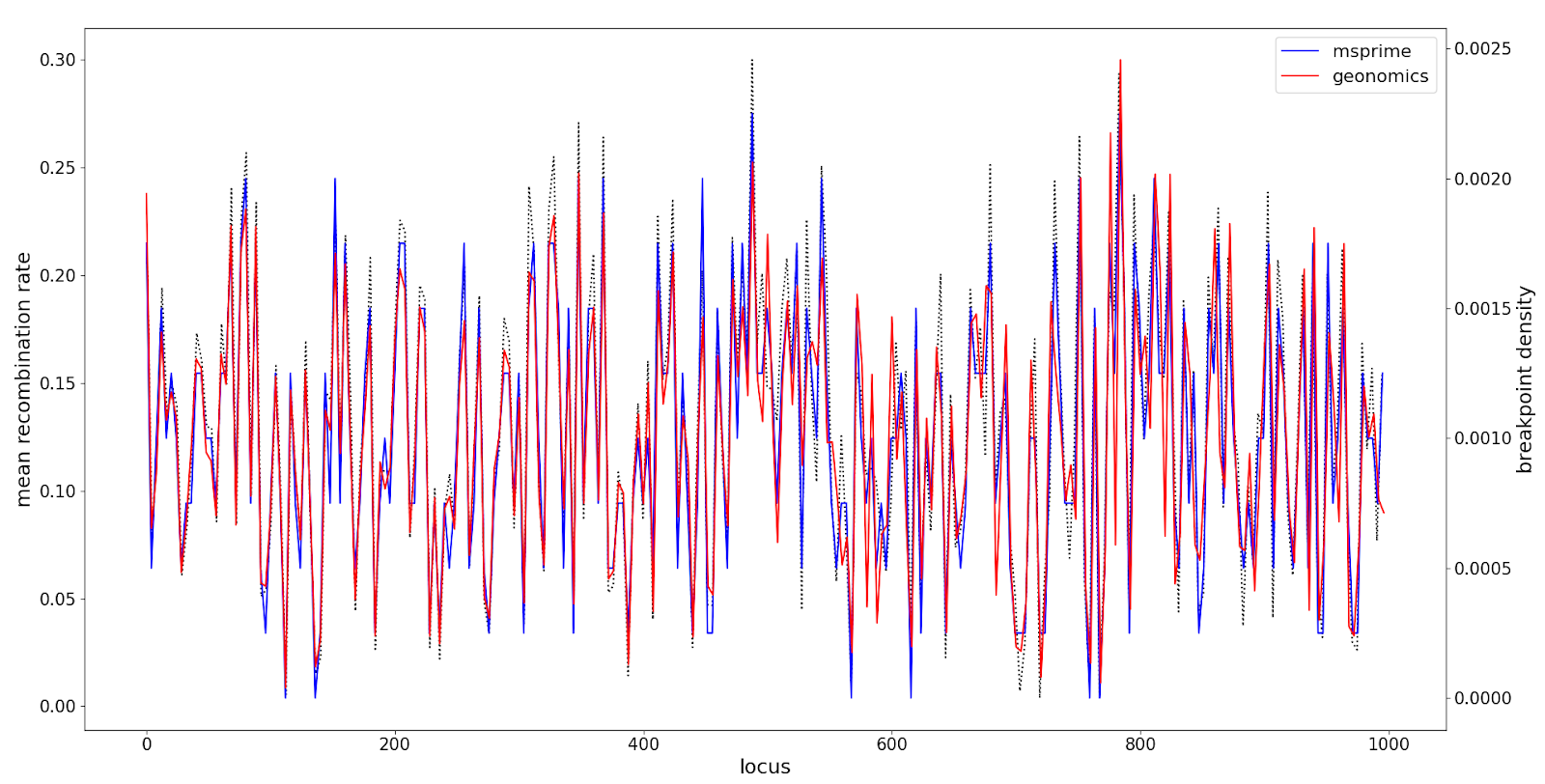
**

**Figure S16:**

*
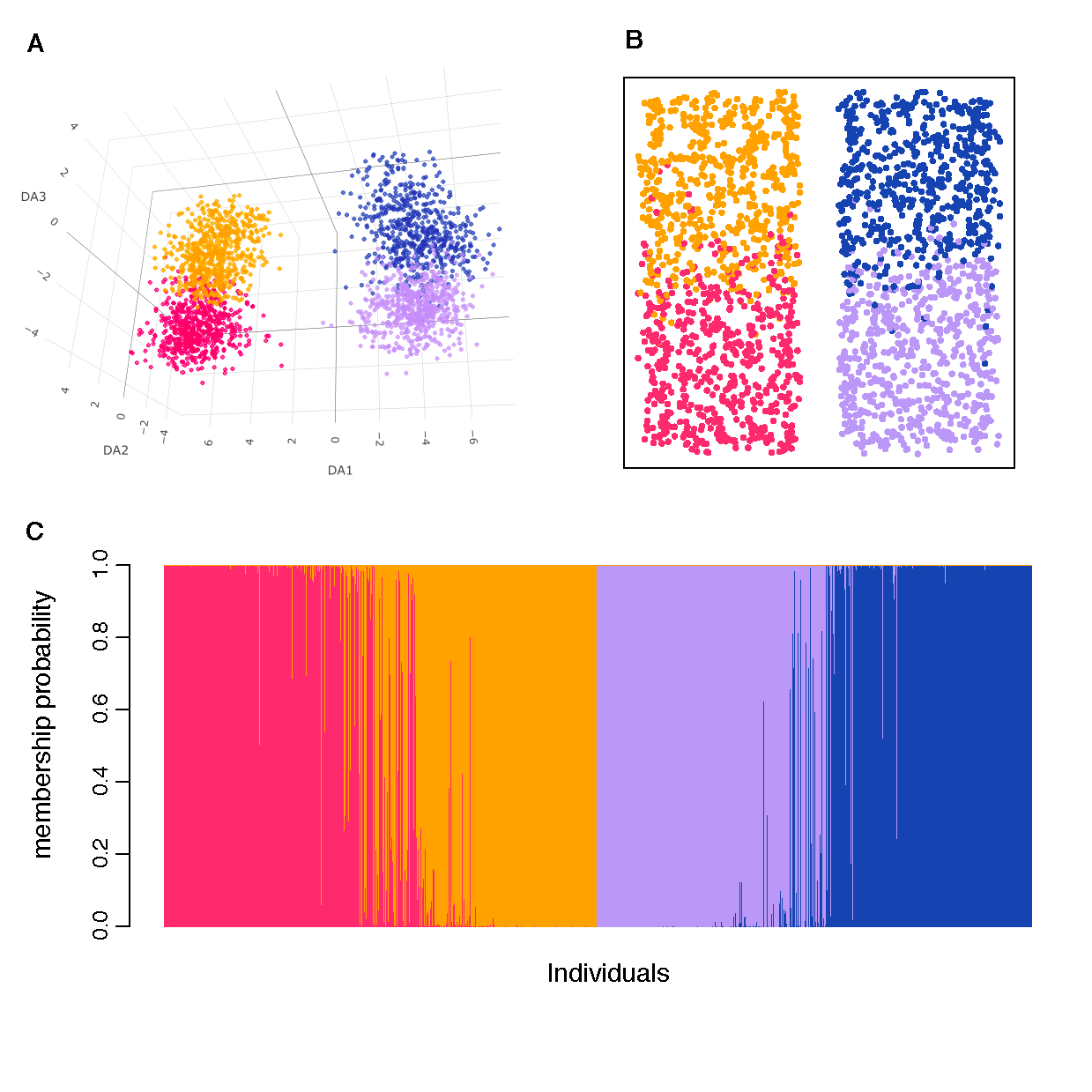
*

**Figure S17:**

*
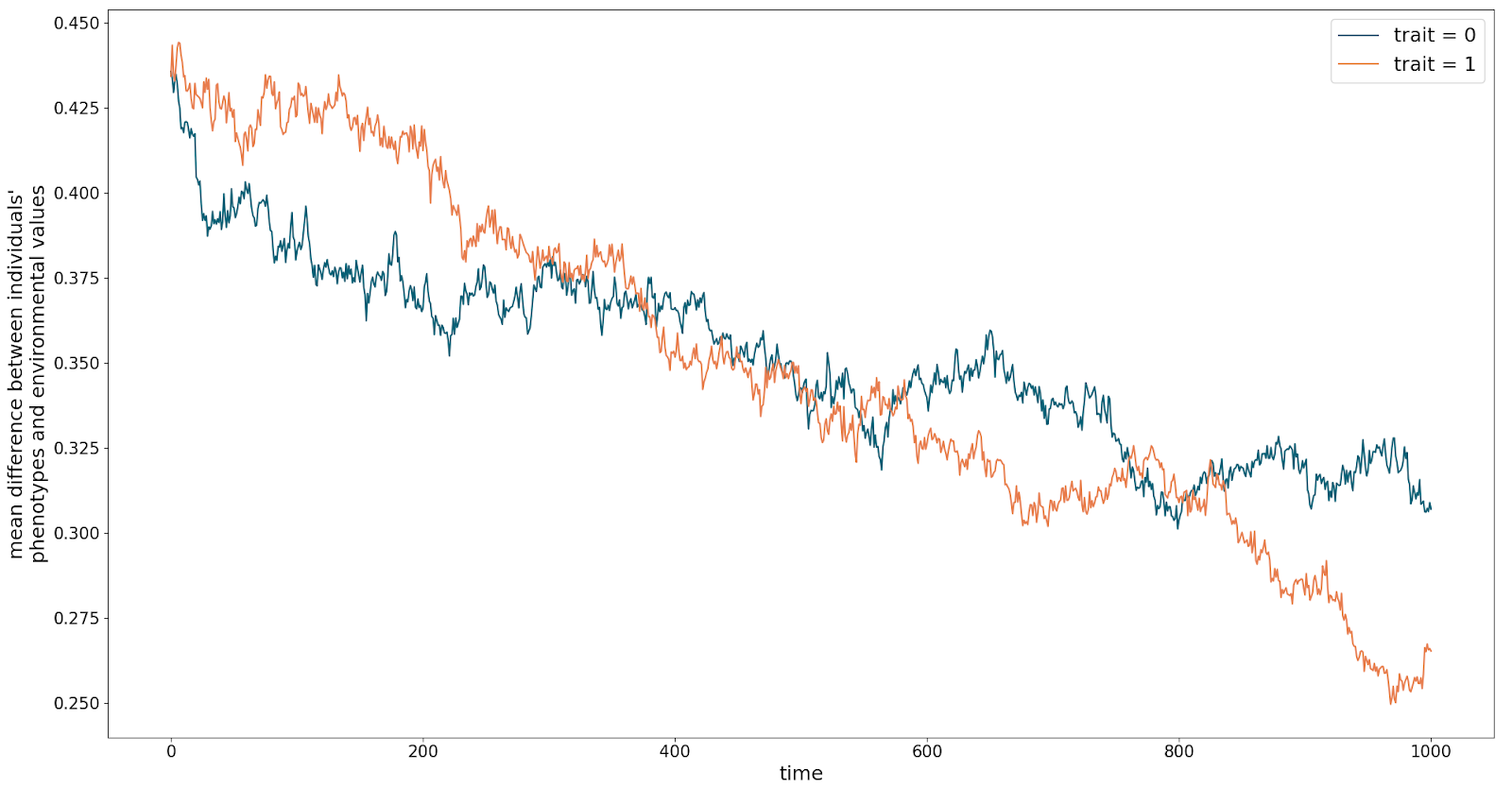
*

**Figure S18:**

*
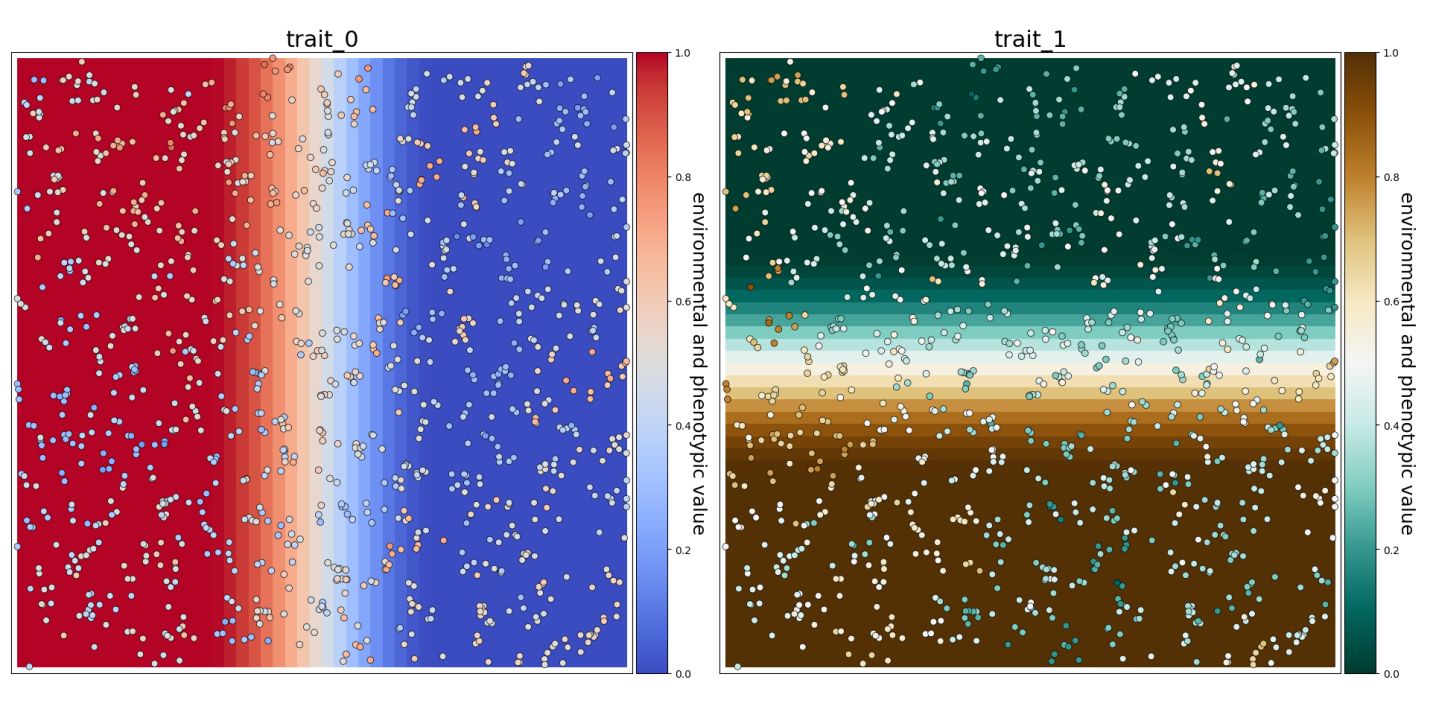
*

**Figure S19:**


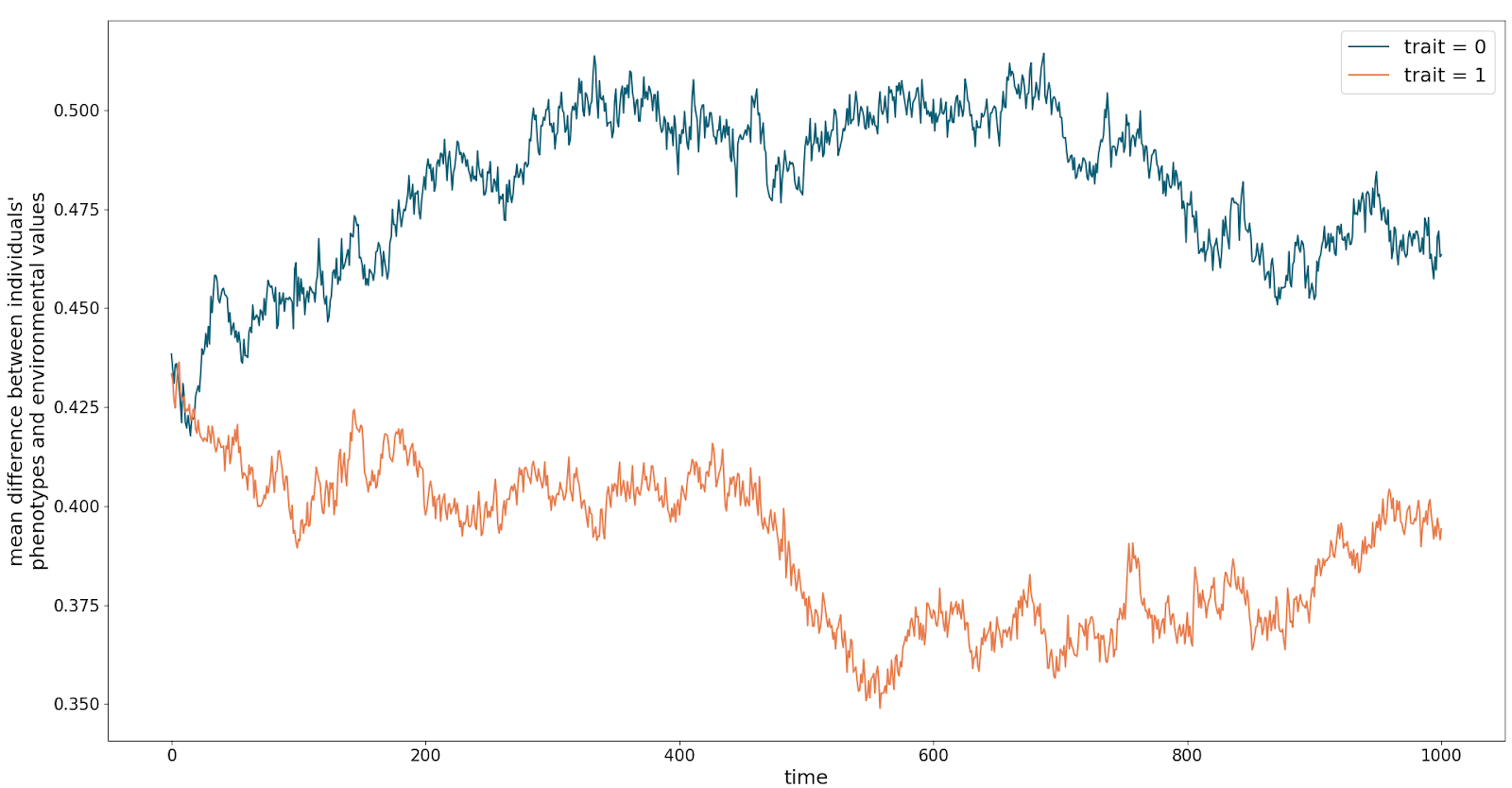

Supplement: msab175_Supplementary_Data [file msab175_supplementary_data.zip › HART_Geonomics_methods_paper_SUPP_R1_5-16-21.docx]
